# Sweet and sour: an update on classic galactosemia

**DOI:** 10.1007/s10545-017-0029-3

**Published:** 2017-03-09

**Authors:** Ana I. Coelho, M. Estela Rubio-Gozalbo, João B. Vicente, Isabel Rivera

**Affiliations:** 1 Department of Pediatrics and Department of Clinical Genetics, Maastricht University Medical Centre, P. Debyelaan 25, PO Box 5800, 6202 AZ Maastricht, The Netherlands; 2 Instituto de Tecnologia Química e Biológica António Xavier, Universidade Nova de Lisboa, Oeiras, Portugal; 3 Metabolism & Genetics Group, Research Institute for Medicines (iMed.ULisboa), Faculty of Pharmacy, Universidade de Lisboa, Lisbon, Portugal; 4 Department of Biochemistry and Human Biology, Faculty of Pharmacy, Universidade de Lisboa, Lisbon, Portugal

## Abstract

Classic galactosemia is a rare inherited disorder of galactose metabolism caused by deficient activity of galactose-1-phosphate uridylyltransferase (GALT), the second enzyme of the Leloir pathway. It presents in the newborn period as a life-threatening disease, whose clinical picture can be resolved by a galactose-restricted diet. The dietary treatment proves, however, insufficient in preventing severe long-term complications, such as cognitive, social and reproductive impairments. Classic galactosemia represents a heavy burden on patients’ and their families’ lives. After its first description in 1908 and despite intense research in the past century, the exact pathogenic mechanisms underlying galactosemia are still not fully understood. Recently, new important insights on molecular and cellular aspects of galactosemia have been gained, and should open new avenues for the development of novel therapeutic strategies. Moreover, an international galactosemia network has been established, which shall act as a platform for expertise and research in galactosemia. Herein are reviewed some of the latest developments in clinical practice and research findings on classic galactosemia, an enigmatic disorder with many unanswered questions warranting dedicated research.

## Introduction

Classic galactosemia (type I galactosemia, OMIM #230400) is caused by deficient activity of galactose-1-phosphate uridylyltransferase (GALT, EC 2.7.7.12), the second enzyme of the main pathway of galactose metabolism, the Leloir pathway, and its prevalence is 1:16,000-60,000 live-births (Ashino et al. 1995; Coss et al. 2013; Fridovich-Keil and Walter 2008). It is an autosomal recessive disorder caused by mutations in the *GALT* gene and over 300 variations have thus far been described (Calderon et al. 2007). Classic galactosemia presents in the neonatal period as a potentially lethal disorder that can lead to chronically debilitating complications (Schweitzer et al. 1993; Waggoner et al. 1990; Waisbren et al. 2012). The only currently available therapeutic strategy is a life-long dietary galactose restriction, which proves insufficient to prevent long-term complications (Bosch et al. 2004b, 2009).

The first description of galactosemia dates back to 1908. Since then, and though a considerable understanding of its molecular, cellular and clinical aspects has been acquired, its exact pathophysiology is not yet fully elucidated.

## Galactose importance in health

Galactose is vital for the human body, exhibiting a broad range of functions, as a key energy source in pre-weaning infants and exerting a crucial structural role, being particularly important for early development (Coelho et al. 2015a).

Galactose is a natural aldohexose that occurs mainly in its d-configuration. It is available as free and bound galactose in complex carbohydrates (such as oligosaccharides and polysaccharides, glycoproteins, and glycolipids). Along with glucose, galactose forms the disaccharide lactose, present in most animal milks and a key energy source in infants.

## Galactose metabolism

The main dietary source of galactose is lactose present in milk and dairy products. After its ingestion, lactose is hydrolyzed in the intestinal lumen by lactase into glucose and galactose. Galactose is transported across the enterocyte brush border membrane by the sodium/glucose active co-transporter SGLT1, and through facilitated diffusion by the GLUT2 transporter across the enterocyte basolateral membrane. Upon entering the blood stream, it is delivered by the portal blood to the liver, the major site of galactose metabolism, where it is internalized by the low-affinity high-capacity GLUT2 (Wood and Trayhurn 2003).

### The main pathway of galactose metabolism

**Fig. 1:**
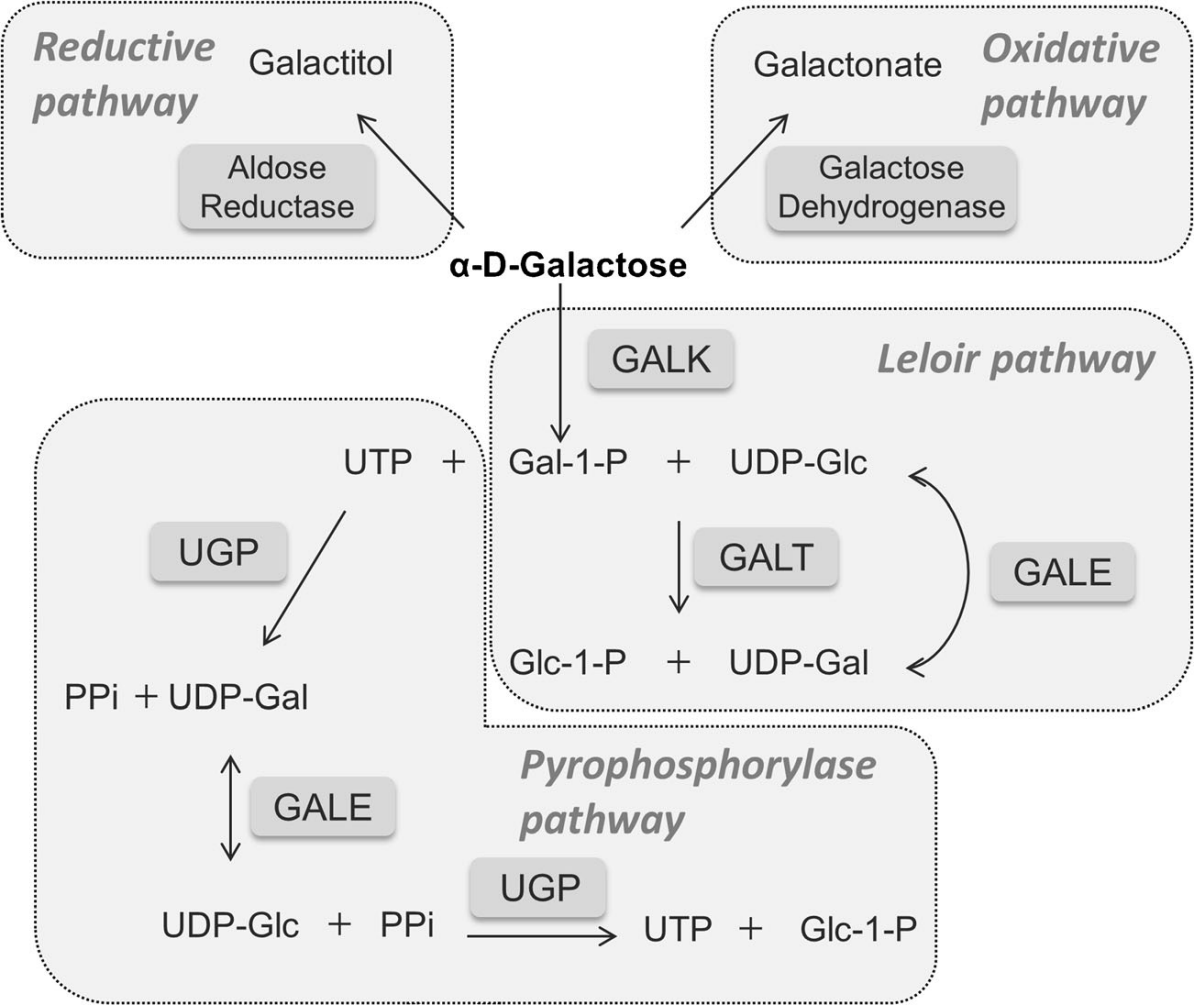
Galactose metabolism. In the Leloir pathway, galactose is converted to glucose-1-phosphate (Glc-1-P) by the action of three consecutive enzymes: galactokinase (GALK1), galactose-1-phosphate uridylyltransferase (GALT), and UDP-galactose 4′-epimerase (GALE). Alternatively, galactose can be reduced to galactitol by aldose reductase, oxidized to galactonate, presumably by galactose dehydrogenase, or be converted into UDP-Glc, via the pyrophosphorylase pathway, by the sequential activities of GALK1, UDP-glucose/galactose pyrophosphorylase (UGP), and GALE. GALE is also responsible for the interconversion of UDP-*N*-acetylgalactosamine to UDP-*N*-acetylglucosamine (not shown). PPi, pyrophosphate

When released from lactose breakdown, galactose is in its beta configuration. Once inside the cells, β-d-galactose is epimerized into its alpha configuration by galactose mutarotase (GALM, EC 5.1.3.3) (Timson and Reece 2003), so that it can enter the Leloir pathway (Fig. 1). This pathway converts α-d-galactose into glucose-1-phosphate (Glc-1-P) by the action of three consecutive enzymes: galactokinase (GALK1) converts α-d-galactose into galactose-1-phosphate (Gal-1-P); galactose-1-phosphate uridylyltransferase (GALT) converts Gal-1-P and uridine diphosphate-glucose (UDP-Glc) into glucose-1-phosphate (Glc-1-P) and uridine diphosphate-galactose (UDP-Gal); and UDP-galactose 4′-epimerase (GALE) is responsible for the interconversion of UDP-Gal to UDP-Glc, as well as of UDP-*N*-acetylgalactosamine to UDP-*N*-acetylglucosamine in mammals. UDP-Glc re-enters the pathway so that further galactose is converted into Glc-1-P and UDP-Gal. The Glc-1-P produced by the Leloir pathway is converted by phosphoglucomutase into glucose-6-phosphate, to be further metabolized via i) the glycolytic pathway; ii) the pentose phosphate pathway; or iii) the gluconeogenic pathway. UDP-Gal, in turn, is the galactose donor for glycosylation reactions.

### Alternative pathways of galactose metabolism

Besides the Leloir pathway, three accessory pathways of galactose metabolism have been described (Fig. 1): i) the reductive pathway, ii) the oxidative pathway, and iii) the pyrophosphorylase pathway.

Galactose reduction occurs through the polyol pathway, consisting of two enzymatic reactions involving the NADPH-dependent aldose reductase (EC 1.1.1.21) and the NAD^+^-dependent sorbitol dehydrogenase (EC 1.1.1.14). Aldose reductase has a broad specificity for monosaccharides, and can metabolize glucose and galactose, respectively yielding sorbitol and galactitol. Although both sugars are similarly processed, sorbitol can be further converted to fructose by sorbitol dehydrogenase, whereas galactitol will accumulate in cells and tissues. In the lens, galactitol buildup produces a hyperosmotic effect, leading to swelling of the cells. Additionally, galactitol production depletes the cell of NADPH, decreasing glutathione reductase activity, and consequently free radicals accumulation, yielding oxidative stress and leading to cell death and cataract formation (Lai et al. 2009; Pintor 2012). Elevated galactitol has also been found in the brain of galactosemic children, where it causes edema (Belman et al. 1986; Berry et al. 2001; Quan-Ma et al. 1966).

Galactose can also be oxidized to galactonate, although the respective pathway is still controversial. It is thought to result from a NAD^+^-dependent galactose dehydrogenase (d-galactose:NAD^+^ oxidoreductase, EC 1.1.1.48) yielding galactonolactone, which is spontaneously or enzymatically converted to galactonate. Galactonate can be directly excreted in this form or further converted via the pentose phosphate pathway into β-keto-d-galactonate, which subsequently undergoes decarboxylation, liberating carbon C1 to form xylulose (Lai and Klapa 2004; Wehrli et al. 1997).

Neither the reductive nor the oxidative pathways involve any of the Leloir enzymes. The pyrophosphorylase pathway, however, depends on GALK1 and GALE, as it involves the GALK1-catalyzed galactose phosphorylation to Gal-1-P followed by galactose incorporation into UDP-Gal catalyzed by UTP-dependent glucose/galactose pyrophosphorylase (UGP, EC 2.7.7.10). UDP-Gal is then epimerized by GALE into UDP-Glc, from which a second pyrophosphorylase reaction generates Glc-1-P and UTP. This pathway has been suggested as a route of endogenous galactose production (Gitzelmann 1969). The major source of endogenous galactose is, however, the lysosomal hydrolysis of galactose-containing glycoproteins, glycolipids and proteoglycans (Berry et al. 1997).

Several studies have provided quantitative evidence for whole body *de novo* galactose synthesis in healthy and galactosemic subjects, estimated between 0.48 and 1.71 mg/kg/h in patients and not influenced by short-term exogenous galactose (Berry et al. 2004; Ning et al. 2000; Schadewaldt 2004; Schadewaldt et al. 2014). Notably, it is considerably higher in infants and children, gradually diminishing until adulthood (Berry et al. 2004; Schadewaldt 2004).

## The molecular biology of classic galactosemia

### GALT protein

**Fig. 2:**
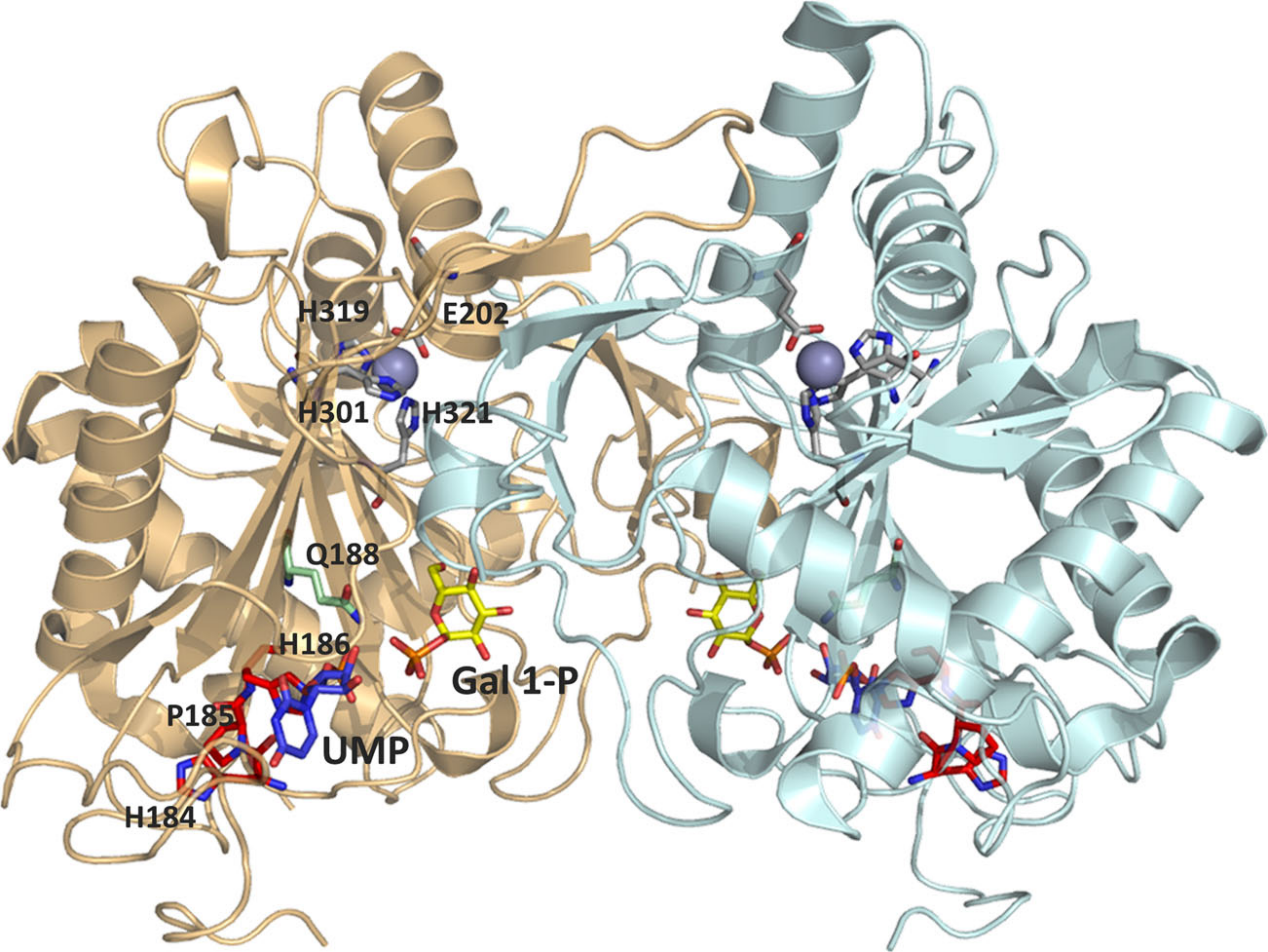
Crystallographic structure of human GALT. Cartoon representation of uridylylated human GALT crystallographic structure obtained in complex with galactose-1-phosphate (PDB code 5IN3). Each monomer is colored either in cyan or light orange. Active site residues H184-P185-H186 in red sticks; covalently linked UMP in blue sticks; galactose 1-phosphate in yellow sticks; residue Q188, responsible for UMP stabilization and site of the most common mutation (Q188R) in classic galactosemia in green sticks; zinc (purple sphere) binding ligands in gray sticks

**Fig. 3:**
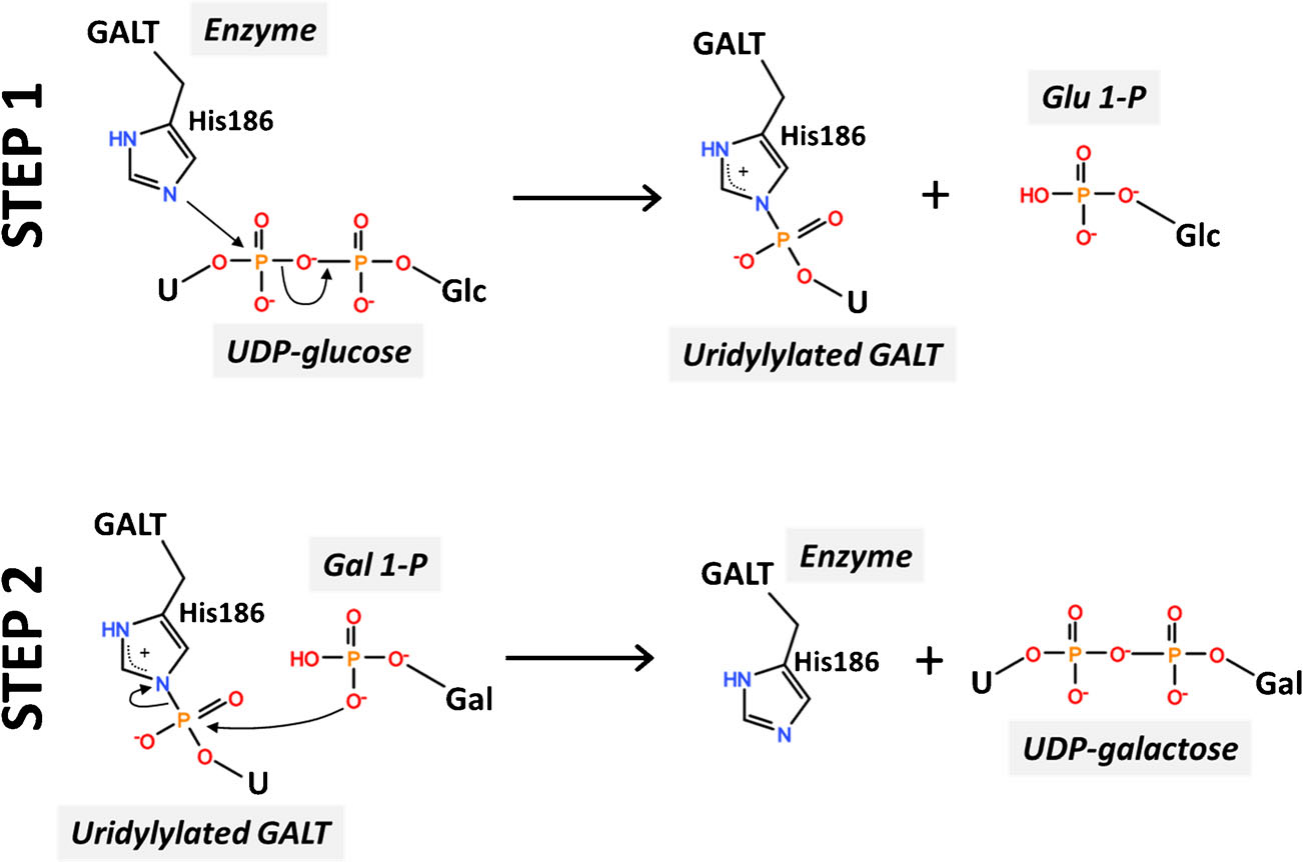
The catalytic mechanism of GALT. In the first step of the reaction, the Nε2 of His186 attacks the α-phosphate of UDP-Glc, releasing Glc-1-P and forming the covalent uridylyl-enzyme intermediate; in the second step, the intermediate reacts with Gal-1-P to produce UDP-Gal

GALT is a ubiquitous enzyme with a remarkable degree of conservation throughout evolution. The first insights into GALT structure came from the X-ray crystallographic structure of *E. coli* GalT, revealing this enzyme is a dimer with two active sites, each formed by amino acids from both subunits (Wedekind et al. 1995). Only recently the crystallographic structure of human GALT was reported (McCorvie et al. 2016) (Fig. 2), confirming the major bacterial GalT structural features, while revealing relevant differences that support a more accurate interpretation and/or prediction of the effect of mutations on GALT structure-function. The human GALT active site contains the H184-P185-H186 sequence, conserved among all known uridylyltransferases (Fig. 2), corresponding in *E. coli* GalT to residues 164–166 (Field et al. 1989; Wedekind et al. 1995). The Glc-1-P binding site is formed by K334, F335, V337, Y339, E340, and Q346 from one chain, along with Q188 and N173 from the other chain. Uridylylation appears to induce a conformational change of GALT, with UMP-GALT presenting a more compact structure than apo-GALT (McCorvie et al. 2016). The fact that both bacterial and human GALT were crystallized in the uridylylated form supports previous proposals of its catalytic mechanism. GALT catalyzes the transfer of an uridyl group from UDP-Glc to Gal-1-P through a double displacement mechanism involving a transiently uridylylated histidine residue (Fig. 3) (Thoden et al. 1997; Wong and Frey 1974a, b). In the first step of this ‘ping-pong’ mechanism, an electron pair from a histidine side-chain imidazole ring in the active site (H186 in human GALT) attacks the UDP-Glc α-phosphate, resulting in a covalently uridylylated intermediate (UMP-enzyme) and releasing Glc-1-P. In the second reaction step, the UMP moiety is displaced from the enzyme by Gal-1-P, thereby regenerating the active site and yielding the second product, UDP-Gal (Wedekind et al. 1996; Wong and Frey 1974b). GALT is a metalloenzyme, although the role of bound metals is likely structural. Each bacterial GalT monomer harbors one zinc ion (bound to C52, C55, H115, H164) and one iron ion (bound to E182, H281, H299, H301). Whereas zinc has been proposed to stabilize the bacterial GalT active site thereby being essential for activity, iron is thought to play a structural role (Geeganage and Frey 1999). The zinc binding residues in *E. coli* GalT are poorly conserved in human GALT. However, the structure of human GALT revealed that a zinc ion is bound to residues E202, H301, H319, H321 (Fig. 2) equivalent to the bacterial GalT iron-binding site. Indeed, and similarly to the proposed role of iron in bacterial GalT, this zinc site was shown to structurally stabilize human GALT and prevent its aggregation (McCorvie et al. 2016).

### GALT gene—clinically relevant variations

The *GALT* gene is located in the 9p13 region, it is arranged into 11 exons spanning ≈ 4.0 kb of genomic DNA (NG_009029.1), and is expressed as a housekeeping gene. Analysis of ∼4 kb of the *GALT* promoter identified three GC-rich Sp1 sites, an imperfect non-palindromic AP-1 site (TCAGTCAG at −126 to −119), a CAAT box, two E-box motifs spaced 23 bp apart (at −146 to −141 bp – CAGGTG and at −117 to −112 bp – CACGTG), and no TATA box sequence (Elsas et al. 2001; Leslie et al. 1992).

An *Alu* repeat motif with evidence for length variation has been identified in intron 10 (Flanagan et al. 2004). It consists of a polyadenine nucleotide repeat with three length variants, (A)17, (A)24, and (A)29 at frequencies of 47.5, 50.0, and 2.5%, respectively. These length variants have defined associations with particular *GALT* alleles. The p.Q188R and p.K285N mutant alleles are in *linkage disequilibrium* with the (A)17 and (A)24 *Alu* variants, respectively, while (A)29 is only found associated with the p.N314D allele (see below) (Flanagan et al. 2004; Fridovich-Keil and Walter 2008).

At present, there are 336 different variations described at the *GALT locus* (available at: http://www.arup.utah.edu/database/GALT/GALT_display.php, last surveyed January 2017) (Calderon et al. 2007). A few of these otherwise rare mutations are common, the most frequent being the c.563A>G transition in exon 6 (CAG → CGG), leading to a substitution of a strictly conserved glutamine to an arginine at residue 188 of human GALT (p.Q188R), two residues after the H184-P185-H186 active site. This mutation accounts for ∼64% of all galactosemic alleles in Caucasian populations, being the most common among people of European ancestry. In Europe, p.Q188R shows a gradient of increasing frequency in the North-Western direction, reaching its highest frequency in Ireland, where it accounts for 92–94% of galactosemic alleles. p.Q188R is the sole variant among the Republic of Ireland Traveller population, where its incidence is estimated to be 1 in 430 (Coss et al. 2013; Flanagan et al. 2010; Suzuki et al. 2001). The consideration that p.Q188R is the most prevalent variation could be biased toward non-Asian origins, since both Chinese and Indian populations (contributing to ∼35% of the World’s population) are largely understudied. p.Q188R was identified in 2.7% of a small cohort of Indian galactosemic patients (Singh et al. 2012), whereas it has never been identified in Chinese or Japanese (or descendant) patients (Ashino et al. 1995; Hirokawa et al. 1999; Suzuki et al. 2001). This mutation is described as giving rise to a non-functional variant. Studies on its bacterial homologue revealed the loss of one H-bond established with UDP-Gal (Geeganage and Frey 1998). In human GALT, p.Q188R has been proposed to cause a destabilization of UMP-GALT (Lai et al. 1999), which was further demonstrated by McCorvie et al. who obtained partially uridylylated (∼8%) p.Q188R GALT after over-night incubation with UDP-Glc (McCorvie et al. 2016). This observation contradicted previous hypotheses of an over-stabilization of UMP-GALT based on homology modeling (Coelho et al. 2014a, b; Facchiano and Marabotti 2010; Marabotti and Facchiano 2005). Moreover, McCorvie and co-workers confirmed the increased aggregation propensity of p.Q188R (Coelho et al. 2014b), proposed therein to result from its lower uridylylation (McCorvie et al. 2016). As compared to uridylylated control p.N314D GALT, p.Q188R also presented a less compact and more elongated shape, similarly to control apo-GALT, reinforcing that some misfolding features might be associated with decreased uridylylation (McCorvie et al. 2016).

Patients homozygous for the p.Q188R variant demonstrate essentially no residual RBC GALT activity, and homozygosity for this allele is often associated with a poor outcome (Robertson et al. 2000; Shield et al. 2000). Transformed lymphoblasts from homozygous p.Q188R patients and heterologous expression in bacterial and yeast systems revealed that this variant displays less than 2% of control activity and poorly alleviates galactose toxicity when expressed in a Δ*galT* ^−^ *E. coli* strain (Coelho et al. 2014b, 2015c; Elsas et al. 1995; Fridovich-Keil and Jinks-Robertson 1993).

The second most common mutation of European origin is c.855G>T in exon 9 (AAG → AAT), leading to a lysine to asparagine substitution at residue 285 of human GALT, p.K285N. This mutation is particularly frequent in countries of Central and Eastern Europe, accounting for 26–34% of galactosemic alleles (Greber-Platzer et al. 1997; Kozák et al. 1999; Lukac-Bajalo et al. 2007; Suzuki et al. 2001; Zekanowski et al. 1999). The Czech, Slovak, Polish, and Austrian galactosemic populations present the highest p.K285N frequencies relatively to other European populations, suggesting a Slavic origin. It has also never been identified in Asian patients (Ashino et al. 1995; Hirokawa et al. 1999; Suzuki et al. 2001). Heterologous expression of this variant revealed essentially null activity (Coelho et al. 2014b; Riehman et al. 2001), and homozygosity in patients is associated with essentially null RBC GALT activity and with a severe clinical phenotype (Zekanowski et al. 1999).

In exon 5, the C→T transition at nucleotide c.404 (TCpG → TTG) leads to a serine to leucine substitution at residue 135 of human GALT, p.S135L. This variant is found almost entirely in individuals of African origin, accounting for approximately 48% of African American and 91% of South African *GALT* mutant alleles (Lai et al. 1996; Manga et al. 1999). Heterologous expression in bacteria or yeast revealed, respectively, less than 0.1 and 2.7% of control activity (Coelho et al. 2014b; Riehman et al. 2001). Notably, p.S135L appears to exhibit tissue specificity: homozygous patients have essentially no GALT activity in RBC, presenting ∼5.5% of control activity in leukocytes, while liver and intestinal mucosa biopsy specimens display ∼10% of normal GALT activity (Lai et al. 1996). Additionally, homozygous patients are capable of converting galactose to CO_2_ at a rate comparable to control subjects and homozygosity for this mutation is often associated with milder clinical outcomes (Berry et al. 1997; Lai and Elsas 2001; Lai et al. 1996; Robertson et al. 2000).

### Duarte and Los Angeles variants

The first known variant of GALT protein is the Duarte variant, named after the city where it was described. The Duarte allele — Duarte-2, D2 or simply D — is biochemically defined as an isoform with a characteristic isoelectric focusing pattern, and a RBC enzyme activity approximately 50% of the control. Heterozygotes for the Duarte allele present 75% of GALT activity, homozygotes present 50% activity, and compound heterozygotes for the Duarte allele and a classic galactosemia allele present 25% activity in RBC (Elsas et al. 1994).

**Fig. 4:**
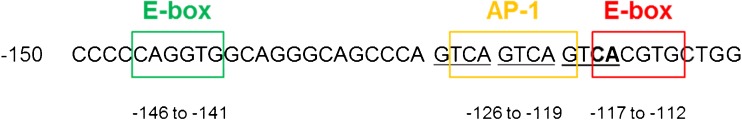
The *GALT* promoter deletion of the Duarte allele. The *GALT* promoter presents an AP-1 site from −126 to −119 bp (TCAGTCAG) and two E-box motifs from −146 to −141 bp (CAGGTG) and from −117 to −112 bp (CACGTG). The −119 to −116 GTCA deletion removes the first two nucleotides (CA, in bold) of the −117 to −112 bp E-box motif. The middle tetrad GTCA fills the deleted CA of the E-box motif; however, the spacing between the two E-box motifs is reduced, and the terminal G of the AP-1 site is removed. Nucleotide +1 is the A of the ATG-translation initiation codon

Later, another variant presenting the same electrophoretic mobility profile as D2 but mildly elevated activity was identified and named as the Los Angeles variant (Duarte-1, D1 or LA) (Langley et al. 1997; Ng et al. 1973). Both variants are now known to be associated with the AAC → GAC transition at nucleotide c.940 in exon 10, leading to an asparagine to aspartate substitution at residue 314 of human GALT (p.N314D). The charge change caused by the substitution of a basic to an acid residue is proposed to be responsible for the resulting specific electrophoretic pattern (Reichardt 1991). The different activities displayed by each variant result from additional base changes: the Los Angeles allele carries p.N314D in *linkage disequilibrium* exclusively with the synonymous mutation c.652C>T (p.L218L), whereas the Duarte allele carries p.N314D in *linkage disequilibrium* with c.378-27G>C (IVS4-27G>C), c.507 + 62G>A (IVS5 + 62G>A), c.508-24G>A (IVS5-24G>A) and a 4-bp deletion in the *GALT* promoter, 116–119 bases upstream the first methionine codon (c.-119_-116delGTCA) (Kozak and Francova 1999; Podskarbi et al. 1996). This deletion removes the first two nucleotides of the −117 to −112 bp E-box motif (CA, located at −117 and −116 bp), which however does not change the E-box motif of CACGTG from −117 to −112 bp, since there are three repeats of GTCA in sequence, and the middle GTCA tetrad fills the deleted CA of the E-box motif (Fig. 4). There is, however, a reduction in the spacing between the two E-box motifs that likely alters *trans*–acting factors binding, thus impairing positive regulation of *GALT* expression. Furthermore, this deletion affects the AP-1 site from −124 to −117 bp (TCAGTCAG), as it removes the terminal guanine of this motif, which can also reduce *GALT* expression. Functional analyses carried out by two independent groups demonstrated that this deletion in the *GALT* promoter is indeed the major factor contributing to the diminished expression and activity of the Duarte variant (Elsas et al. 2001; Trbušek et al. 2001).

At present, p.N314D is a common allele with a frequency of approximately 11% in European populations and lower frequencies in other populations, with an 8.3% pan-ethnic frequency (Carney et al. 2009; Suzuki et al. 2001). Interestingly, a study on the origin and distribution of the Duarte allele suggested that p.D314 is in fact the ancestral allele and that p.N314 only arose early in human evolution once humans migrated out of Africa (Carney et al. 2009).

Functional and structural characterization of the p.N314D variant confirmed that, *in vitro*, it displays enzymatic activity essentially identical to control, although with impaired conformational stability (Coelho et al. 2014b). This variant was recently used for the first description of the crystallographic structure of human GALT (McCorvie et al. 2016).

## Biochemical features and follow-up

Severe impairment of GALT activity results in the accumulation of metabolites such as galactose, Gal-1-P, galactitol, and galactonate, and in deficiency of UDP-Gal and UDP-Glc.

Galactose only differs from glucose in the hydroxyl moiety configuration at the carbon-4 position. However, galactose is less stable and more susceptible to the formation of nonspecific glycoconjugates (Decombaz et al. 2011). The human body is able to metabolize large galactose amounts, as evidenced by its rapid clearance from blood: 50% of radiolabeled galactose is found in glucose pools within 30 min after intravenous administration. For nursing infants, galactose conversion to glucose is crucial to maintain euglycemia, since 40% of calories derive from lactose hydrolysis into galactose and glucose (Coelho et al. 2015a). In contrast to healthy infants, galactosemic newborns accumulate galactose in the blood, as well as in other cells and tissues, and they present high urinary galactose concentrations, whereas blood glucose levels can fall to hypoglycemic levels. Upon initiation of dietary galactose restriction—the current standard of care—blood galactose levels fall quickly, but always remain elevated. Additionally, patients also present high RBC Gal-1-P concentrations which, despite falling dramatically upon diet implementation, never normalize (Gitzelmann 1995). Gal-1-P accumulation has been widely pointed out as a key pathogenic agent (Gitzelmann 1995; Lai et al. 2009; Leslie 2003; Tang et al. 2010). Gal-1-P has been described to inhibit *in vitro* enzymes involved in carbohydrate metabolism, such as UDP-glucose pyrophosphorylase, glucose-6-phosphatase, glucose-6-phosphate dehydrogenase, phosphoglucomutase, and glycogen phosphorylase (Gitzelmann 1995). Additionally, Gal-1-P has been described to inhibit inositol monophosphatase, which could lead to a reduced inositol pool. Brain autopsy of two newborn patients showed a reduction of up to 80% free inositol (Ins) and phosphatidyl-inositol (PtdIns) comparatively with healthy controls, and rats with a high-galactose diet exhibited a reduction of up to 30% of free Ins and PtdIns (Bhat 2003). There is also evidence that high levels of Gal-1-P inhibit galactosyltrans-ferases, which may disturb glycosylation (Charlwood et al. 1998; Jaeken et al. 1992). Additionally, UDP-Gal and UDP-Glc levels have been found decreased in a number of studies (Coss et al. 2012, 2014; Lai et al. 2003a; Ng et al. 1989). These UDP-hexoses are the galactose and glucose donors in glycosylation reactions, and several studies have indeed reported glycosylation abnormalities in classic galactosemia (Charlwood et al. 1998; Coman et al. 2010; Coss et al. 2012, 2014; Lebea and Pretorius 2005; Liu et al. 2012; Maratha et al. 2016; Petry 1991; Sturiale et al. 2005).

High galactitol levels have been found in blood, tissues, and urine of galactosemic patients (Berry et al. 2001; Jakobs et al. 1995; Palmieri et al. 1999). When dietary treatment is implemented, most galactitol is cleared via urine, but does not entirely disappear, likely because of endogenous galactose production.

Galactonate has been detected in urine and in several tissues of galactosemic patients. However, galactonate in patients’ plasma is below the detection level, and is not elevated in the RBC of all galactosemic patients (Ning and Segal 2000; Wehrli et al. 1997; Yager et al. 2003).

## Diagnosis

### Acute clinical presentation and diagnosis

Infants with classic galactosemia generally appear asymptomatic at birth. However, after a few days of galactose ingestion through breast and/or formula feeding, children start developing life-threatening symptoms that, if undiagnosed and untreated, may lead to death. Initial symptoms include poor feeding with poor weight gain, vomiting and diarrhea, hepatocellular damage, lethargy, and hypotonia. Progression of this acute neonatal toxicity syndrome may include the development of Gram negative sepsis, cataracts, and *pseudotumor cerebri* causing a bulging fontanel (Berry and Walter 2012).

There are several tests to diagnose classic galactosemia. Screening for reducing substances in urine can be informative; however, it is not sensitive or specific, leading to false positives due to fructosuria, lactosuria (from intestinal lactase deficiency), or to conditions that impair blood galactose clearance, such as severe liver disease or antibiotic treatment. In contrast, if the child is on intravenous feeding, galactosuria may no longer be present, thus leading to false negatives (Berry and Walter 2012; Bosch 2006).

A more specific approach is the measurement of galactose metabolites, such as galactose, Gal-1-P and/or galactitol, in blood and/or urine. Galactosemic patients present invariably high RBC Gal-1-P levels. However, benign variants can also originate increased Gal-1-P levels and diagnosis should not be done based exclusively on it (Berry 2012; Berry and Walter 2012). Hemolysate Gal-1-P can be measured by different methods: spectrophotometric coupled enzymatic assay, isotope-dilution gas chromatography–mass spectrometry (GC/MS), or tandem MS (MS/MS) (Chen et al. 2002a; Jensen et al. 2001; Schadewaldt et al. 2003). Another approach is the Paigen test, which quantifies total galactose through a microbiological assay which is, however, not suitable for automation and is sensitive to antibiotic therapy taken by newborns or their mothers (Beutler 1991). Nursing infants with galactosemia also present high galactitol amounts in their urine and plasma, which stay higher than in controls, even upon diet implementation. Galactitol in urine can be measured by nuclear magnetic resonance (NMR) or GC/MS, whereas in RBC GC/MS is required due to its higher sensitivity (Chen et al. 2002b; Palmieri et al. 1999; Wehrli et al. 1997; Yager et al. 2003, 2006).

The gold standard for diagnosis is the GALT activity measurement in RBC, which can be done by the semi-quantitative Beutler fluorescent spot test or by a preferable quantitative assay. It can be done indirectly by a coupled enzymatic assay, or directly by quantifying radioactively labeled Gal-1-P conversion to UDP-Gal or, alternatively, by quantifying unlabeled substrates and products by HPLC (Cuthbert et al. 2008; Lindhout et al. 2010). Classic galactosemic patients usually present undetectable or less than 1% of control GALT activity (Elsas 2010). RBC GALT activity analysis is, however, not suitable for children that have been subjected to blood transfusion within the last 3 to 4 months, since a false-negative might result.

*GALT* mutational analysis is currently available in many laboratories for the most common variations, and has been used in some newborn screening programs to refine the screening process. A negative result does not exclude the disease, and full gene sequencing, including deep intronic regions, may be required (Coelho et al. 2015b; Wadelius et al. 1993).

## Newborn screening

Neonatal screening for classic galactosemia has been debated for years, but no consensus has been reached. The principles originally articulated by Wilson and Jungner for screening and later expanded by Pollitt et al. have been accepted as the criteria for neonatal screening (Clarke 2005; Pollitt et al. 1997). Galactosemia has been excluded from some newborn screening programs based on two major arguments: it can be diagnosed clinically and long-term complications still develop despite early treatment. Despite Pollitt et al. recognizing that effective treatment availability was not an absolute prerequisite for a disease to be included in newborn screening programs, galactosemia-specific screening was still not recommended. Nevertheless, there were recommendations for all samples with increased phenylalanine to be screened for galactosemia as a secondary test. A later study compared 139 children with metabolic diseases identified by neonatal screening (17 galactosemic) to 124 children identified on the basis of clinical symptoms (9 galactosemic), and concluded that, despite the similar rate of hospitalization, 47% of the clinically identified children had mental retardation compared to only 14% of those identified by newborn screening, and that parental stress tended to be greater in families of children identified clinically (Waisbren et al. 2002).

Newborn screening for galactosemia is included in several European countries—Austria, Germany, Hungary, Ireland, Sweden, Switzerland, The Netherlands—while others—Turkey, Italy, and Belgium—present pilot programs (EGS 2003; Karadag et al. 2013; Ohlsson et al. 2012). Ireland presents a high classic galactosemia incidence (1:16,476), especially in the Traveller population (1:430), and newborn screening for classic galactosemia has been performed since 1972 (Coss et al. 2013). Irish newborns undergo screening within 72 to 120 h after birth, while all high-risk Traveller neonates are evaluated at day 1 or 2. On the other hand, Sweden presents a relatively low frequency (1:100,000) and yet newborn screening program includes galactosemia (Ohlsson et al. 2012). Other countries, such as France and Portugal do not offer neonatal screening and some, like Norway, Denmark, and Scotland, have stopped their programs (EGS 2003; Hansen and Lie 1988; Lund et al. 2012; Shah et al. 2001). In the USA, screening for galactosemia is currently offered in all states, and in Canada only two out of ten provinces offer universal screening (Fridovich-Keil and Walter 2008; Shah et al. 2001).

## Standard of care

Lifelong dietary restriction of galactose has been the therapeutic basis for classic galactosemia since Mason and Turner described in 1935 how removing galactose from the diet eliminated the acute clinical presentation.

After the initial symptoms and upon the first suspicion of galactosemia, patients should be put immediately on a galactose-restricted diet (Berry and Walter 2012). Commercial infant formulas appropriate for use in galactosemia management include elemental and soy formulas. Despite elemental formulas containing no galactose, there is insufficient evidence that supports their added value comparatively with soy formulas. In fact, the latter are most often recommended, except for premature infants, in which case an elemental formula is preferable (Van Calcar et al. 2014a).

With the introduction of solid foods, some galactose is inevitably introduced into the diet. While there is consensus regarding the restriction of most dairy products, cheese consumption has always been a matter of debate. Some studies have been developed to elucidate the galactose content in several cheeses, revealing that most mature cheeses are adequate for galactosemia diet (Portnoi and MacDonald 2009; Portnoi and MacDonald 2011; Portnoi and MacDonald 2016; Van Calcar et al. 2014b). Galactose is also naturally found in cereals, offal meats, pulses, fruits, and vegetables; their galactose content, however, is considerably lower than in lactose-rich foods (Gropper et al. 2000; Gross et al. 1995; Gross and Acosta 1991; Kim et al. 2007; Van Calcar et al. 2014b). In addition, galactose can also be found in its complex form, which is presumably hydrolyzed in the gastrointestinal tract, rendering galactose available for absorption. However, presently it is considered that most galactose is actually not available for digestion (Van Calcar et al. 2014a).

Concerning galactosemia outcome, the significance of non-dairy foods to the total galactose intake has long been questioned. Berry et al. demonstrated that a lactose-free diet highly enriched in fruits and vegetables resulted in a mean galactose intake of 54 mg per day, and further demonstrated that the daily ingestion of 200 mg of fruit-derived galactose (50 mg of pure galactose in fruit juice 4 times per day) during 3 weeks had no impact on RBC Gal-1-P values and relatively little effect on urinary galactitol levels, despite the patients having null RBC GALT activity (Berry et al. 1993). In another study, Bosch et al. demonstrated that galactose supplementation for 6 weeks up to a maximum of 600 mg per day—corresponding to the amount of galactose in 7 kg of apples, 2.5 kg of tomatoes or 12 kg of peas—did not result in any physical, ophthalmological, or biochemical abnormalities (Bosch et al. 2004a). These data suggest that reducing the daily dietary galactose intake by restricting fruit and vegetables is negligible and, in the short-term, does not seem to have a significant impact on the clinical outcome. In a retrospective study, Hughes et al. reported no difference in long-term complications of siblings from 14 families (total of 30 subjects) with lower (<20 mg/day) and higher galactose intake (>20 mg/day) (Hughes et al. 2009). Another study reported that the ingestion of galactose, gradually increased from 300 to 4000 mg/day for a period of 16 weeks, improved the abnormalities in plasma IgG *N*-glycan profiles (Coss et al. 2012). Furthermore, the realization that galactosemic patients endogenously synthesize galactose to an extent that far exceeds that from non-dairy foods has minimized this concern. Accordingly, some countries have liberalized the therapy, currently advising a lactose-free diet (Bosch et al. 2004a; Van Calcar et al. 2014a). The international clinical guideline for the management of classic galactosemia recommends a life-long galactose-restricted diet that only eliminates sources of lactose and galactose from dairy products, allowing mature cheeses, caseinates, and non-milk foods (Welling et al. 2016).

## Pathophysiology and long-term outcome

While the success of early treatment on the acute neonatal symptoms is unquestioned, the long-term outcome can be extremely disappointing since many patients develop burdensome complications.

### Cognitive impairment

One of the most frequent and well-established long-term complications is cognitive impairment. Across most studies, IQ standard scores have been found to be in the low average (85–100) to borderline-low (<85) range, with considerable inter-individual variability (Antshel et al. 2004; Doyle et al. 2010; Kaufman et al. 1995; Potter et al. 2008; Waggoner et al. 1990; Waisbren et al. 1983). Motor disturbances, such as coordination, gait, balance, fine motor tremors, and severe ataxia are quite frequent, as well as memory, speech, and language problems (Antshel et al. 2004; Hughes et al. 2009; Kaufman et al. 1995; Potter 2011; Potter et al. 2008, 2013; Robertson et al. 2000; Schweitzer et al. 1993; Timmers et al. 2011, 2012; Waggoner et al. 1990; Waisbren et al. 1983, 2012). Speech and language impairment has been estimated to affect 38–88% of patients and cannot be solely explained by lower cognitive abilities in general (Hughes et al. 2009; Potter et al. 2008; Robertson et al. 2000; Schweitzer et al. 1993; Timmers et al. 2011, 2012; Waggoner et al. 1990; Waisbren et al. 1983). Expressive language is mainly affected, with receptive language or comprehension being relatively preserved (Potter et al. 2008; Timmers et al. 2011). Patients exhibit speech motor abnormalities such as childhood dyspraxia of speech and voice dysfunction (Potter 2011; Potter et al. 2013), besides impaired cognitive planning of language at several stages, from conceptualization to lexical and syntactic planning (Timmers et al. 2012), showing altered neural activity and connectivity patterns (Timmers et al. 2015a).

Studies on galactosemic patients’ brains have reported structural abnormalities, such as cerebral and cerebellar atrophy, and white matter abnormalities possibly due to altered myelination (Crome 1962; Haberland et al. 1971; Hughes et al. 2009; Koch et al. 1992; Krabbi et al. 2011; Lo et al. 1984; Nelson et al. 1992; Wang et al. 2001). A recent study on the white matter microstructure in galactosemic patients revealed increased neurite dispersion (i.e., less organized axons) and lower neurite density (Timmers et al. 2015b). Impaired galactosylation of glycoconjugates in the brain has been suggested as the underlying pathogenic mechanism (Coss et al. 2012; Petry 1991).

Visual and perceptual impairments also occur in galactosemic patients and seem to be independent of the IQ (Antshel et al. 2004; Kaufman et al. 1995; Schweitzer et al. 1993). Handwriting, reading, and difficulties in mathematics are also common, and galactosemic children are often academically retained (Antshel et al. 2004; Schweitzer et al. 1993). School achievements are below the grades reached by healthy siblings and/or parents. Whereas a comprehensive multidisciplinary study on galactosemic adults reported an average schooling of 1 to 2 years of college, with an occupational level of skilled manual laborer (Waisbren et al. 2012), another revealed that many patients lacked educational qualifications and were unemployed (Bhat et al. 2005). Patients are often described as shy and reserved but generally do not present behavioral dysregulation (Antshel et al. 2004), and are single and live with their parents, as only a minority is able to build up strong partnerships outside the family core (Waisbren et al. 2012). Approximately 40% of patients recognize that galactosemia impairs their relationships with other people (Hoffmann et al. 2012). Bosch and colleagues reported that galactosemia is seen as a burden by a significant number of patients (39%), with many feeling different because of the disease (34%), and believing they are not well understood (22%). Furthermore, many parents feel the disorder influenced their contact with the child (73%) and that taking care of a galactosemic child represents a burden (60%), despite raising their child the same way as their healthy children (69 and 77%, respectively) (Bosch et al. 2004b). This study concluded that galactosemia negatively influences the health-related quality of life (HRQoL) on the communication, social and, most strikingly, cognitive domains. In another study, Bosch et al. compared the course of life of galactosemic patients with that of the general population and with phenylketonuric patients (Bosch et al. 2009). Galactosemic patients achieved fewer social and psychosexual developmental milestones as compared to healthy or phenylketonuric controls, suggesting that these differences result from specific complications of classic galactosemia and not from the burden of a chronic disease or lifelong dietary restrictions. The social and psychosexual development milestones of men studied in a small cohort appeared severely delayed, leading to the suggestion that early intervention (e.g., social skills training) might improve the patients’ psychosocial competences (Gubbels et al. 2011).

### Bone health

Because of their galactose-restricted diet, patients are at risk for nutritional deficiencies, particularly regarding calcium, since dairy products are considered its best source for their high calcium content in casein-micelles (Lewis 2011). Decreased height and growth rate have been reported. However, growth often continues through the late teens, so that the target height can be reached by children who grow beyond the age of 18 (Panis et al. 2007; Waggoner et al. 1990). Treated galactosemic patients are at risk for diminished bone mineral density (BMD) (Batey et al. 2013; Kaufman et al. 1993; Panis et al. 2004; Rubio-Gozalbo 2002; van Erven et al. 2017). A patient group with no evidence of nutritional deficiencies exhibited decreased levels of bone formation and resorption markers (Panis et al. 2004). Since carboxylated osteocalcin is a marker for the vitamin K nutritional status, Panis et al. developed a 2-year randomized clinical trial to investigate whether vitamin K, given in combination with calcium and vitamin D, could play a role in the pathophysiology of this complication. In fact, they found a statistically significant increase in osteocalcin carboxylation in both prepubertal and pubertal children receiving supplementation (Panis et al. 2006).

Batey et al. reported that out of 32 evaluated adult patients, only 10% were followed by a nutritionist, suggesting nutritional counseling as a strategy to optimize bone accrual during adolescence and to maintain bone mass during adulthood (Batey et al. 2013). Nevertheless, several studies have excluded major nutritional deficiencies, and concluded that other intrinsic factors must be involved (Batey et al. 2013; Kaufman et al. 1993; Panis et al. 2005, 2006).

### Gonadal impairment

The most common complication reported for girls and women with classic galactosemia is primary ovarian insufficiency (POI), with an incidence above 80% (Berry 2008; Fridovich-Keil et al. 2011; Kaufman et al. 1981; Rubio-Gozalbo et al. 2010; Waggoner et al. 1990). POI clinical manifestations range from absent or delayed pubertal development, primary amenorrhea, secondary amenorrhea or oligomenorrhea, and premature menopause. Many female patients do not spontaneously reach puberty, which has to be induced to reach a normal pubertal development and prevent sequelae (Gubbels et al. 2008; Spencer et al. 2013). The proposed pathogenic mechanisms include ovarian damage due to elevated Gal-1-P and galactitol; UDP-Gal deficiency causing aberrant glycosylation of glycoconjugates involved in ovarian function; increased apoptosis of maturing follicles, with accelerated follicle atresia; and possible abnormalities of the immune system, such as unrecognized auto-ovarian antibodies (Forges et al. 2006; Lai et al. 2003b; Liu et al. 2000; Rubio-Gozalbo et al. 2010). Two neonates have been reported to have morphologically normal ovaries and abundant oocytes (Levy 1996; Levy et al. 1984), whereas young adult females’ ovaries have been reported to have a severely decreased number of normal primordial follicles (Rubio-Gozalbo et al. 2010). Follicle-stimulating hormone (FSH)—an indirect marker of ovarian reserve—has been found elevated in patients very early in life up to the onset of puberty (4 months to 12 years) (Berry 2008; Fridovich-Keil et al. 2011; Rubio-Gozalbo et al. 2010; Sanders et al. 2009). Anti-Mullerian hormone (AMH)—a direct marker for ovarian reserve that is produced by granulosa cells in the developing ovarian follicle—has been found low in female patients (Sanders et al. 2009; Spencer et al. 2013). However, low AMH levels in galactosemia patients might be not only due to follicle depletion, but might also reflect an impaired follicle maturation (Rubio-Gozalbo et al. 2010). Indeed, extremely low AMH levels do not preclude spontaneous pregnancy (Gubbels et al. 2009). In fact, spontaneous pregnancies occur (Briones et al. 2001; De Jongh et al. 1999; Gubbels et al. 2009; Kimonis 2001; Ohlsson et al. 2007; Roe et al. 1971; Samuels et al. 1976; Tedesco et al. 1972). And, whereas in the past women were told to be infertile, presently women are informed that spontaneous pregnancies may occur. The term subfertility is more accurate than infertility. Nevertheless, subfertility remains a major concern for patients and parents, and physicians are often asked about fertility preservation options (Spencer et al. 2013; van Erven et al. 2013).

Few studies have examined the male reproductive system and, until recently, male fertility was believed not to be impaired (Gubbels et al. 2013; Rubio-Gozalbo et al. 2010). A study on cryptorchidism in galactosemic individuals revealed a 25% prevalence (three of the 12 males), compared with ≤1% in the healthy age-matched population (Rubio-Gozalbo et al. 2006). A more recent investigation on the reproductive system of 26 galactosemic patients demonstrated an increased prevalence of cryptorchidism (11.6%), with low semen volume, and lower testosterone, inhibin B and sperm concentrations than in control subjects—although within the normal range on average—which might indicate mild defects in Sertoli and Leydig cell functions (Gubbels et al. 2013). With the exception of cryptorchidism, these abnormalities are expected to have little impact on fertility (Gubbels et al. 2013; Rubio-Gozalbo et al. 2006). Besides the three fathered pregnancies in the literature, there are no data on paternity in galactosemic patients other than anecdotal cases which may be related to a publication bias and to difficulty in documenting paternity, or to the known social interaction problems and delayed psychosocial development documented in these men (Gubbels et al. 2013; Waisbren et al. 2012).

## Models of classic galactosemia: toward the understanding of the underlying pathophysiology

Different cellular and animal models have been developed to understand the pathogenic mechanisms of classic galactosemia.

### Yeast model

*Saccharomyces cerevisiae* deleted in the *GAL7* gene (the yeast orthologue of human *GALT*) shows a growth arrest upon galactose addition to the medium despite the presence of other carbon sources (Douglas and Hawthorne 1964, 1966). Using GALT-deficient yeast, Fridovich-Keil’s group has studied several human *GALT* mutations (Crews et al. 2000; Fridovich-Keil and Jinks-Robertson 1993; Fridovich-Keil et al. 1995; Mumma et al. 2008; Ross et al. 2004; Wells and Fridovich-Keil 1997). Furthermore, the yeast model has been used to provide new insights on the metabolic role of each of the Leloir pathway enzymes and galactose metabolites (Riehman et al. 2001; Ross et al. 2004). Interestingly, *GAL1*- (orthologue for the human *GALK1* gene) deficient yeast does not cease to grow upon galactose addition to the growth medium (Ross et al. 2004; Slepak et al. 2005). Additionally, the yeast model of classic galactosemia has been used to study gene expression upon galactose exposure (De-Souza et al. 2014; Slepak et al. 2005), revealing that galactose induces metabolic and endoplasmic reticulum (ER) stress and triggers the unfolded protein response (UPR).

### Bacterial model

Bacterial expression systems have been used to study several human *GALT* mutations, by expressing recombinant human GALT variants (Coelho et al. 2014b; Lai and Elsas 2001; Lai et al. 1999). Most recombinant human GALT variants displayed a severely impaired catalytic activity and/or decreased stability, establishing GALT misfolding and/or aggregation as the pathogenic mechanism underlying several variants, leading to the classification of classic galactosemia as a conformational disorder (Coelho et al. 2014b; McCorvie et al. 2013).

A prokaryotic model with a deletion of the endogenous *galT* gene has been employed to evaluate the functional impact of several human *GALT* mutations in a cellular context. Expression of the p.Q188R, p.K285N, p.G175D, and p.P185S variants failed to alleviate galactose toxicity (Coelho et al. 2015c).

### Mouse model

Two decades ago, the first animal model of classic galactosemia—a *Galt*-knockout mouse—was developed (Leslie et al. 1996), displaying a complete loss of Galt activity and accumulating Gal-1-P at levels comparable to those in galactosemic humans. However, the mouse was described to remain healthy despite dietary exposure to galactose. More recently, another Galt-deficient mouse model was developed by Lai’s group, using a *Galt* gene-trapping approach, which exhibited null Galt activity and Gal-1-P accumulation in RBC (Tang et al. 2014). Despite some resistance to galactose toxicity, expanded characterization of the model revealed subtle phenotypic differences to wild-type mice. Histological examination of galactose-fed pups revealed hepatic and brain (cerebral and cerebellar) alterations. Additionally, excess galactose also led to manifestation of oxidative stress in mutant pups (lower GSH/GSSG ratio) comparatively to wild-type or non-challenged mutant pups. In the long-term, some degree of growth and fertility impairments also became evident. The newborn intoxicated pups that survived the galactose insult manifested a decreased growth rate and weight, even when normal chow resumed after the weaning period ended, that was only recovered with puberty. Additionally, mutant mice exhibited a smaller litter size and a longer time to achieve pregnancy, suggestive of subfertility.

### Fruit fly model

A Galt-deficient *Drosophila melanogaster* model has also been developed, which showed a null GALT activity and Gal-1-P accumulation (Kushner et al. 2010). If exposed to galactose, these flies succumbed during development; however, they lived when maintained on a galactose-restricted diet. In the long-term, Galt-deficient adult flies under a galactose-restricted diet demonstrated an impaired negative geotaxic response, suggesting the development of movement impairments. Both galactose lethality and motor impairments could be rescued by the expression of a wild-type human *GALT* transgene (accounting for ≈ 4% of wild-type GALT activity). A more recent study has shown that GALT deficiency results in structural synaptic overelaboration and glycosylation abnormalities of the flies neuromuscular junction (NMJ), namely reductions in the galactosyl, N-acetylgalactosamine, and fucosylated moieties (Jumbo-Lucioni et al. 2014a). Mutant flies on restricted-galactose and high-galactose diets showed identical movement impairments and overelaborated NMJ architecture, reinforcing the notion that classic galactosemia’s long-term phenotype is independent of dietary galactose. Additionally, the Wnt trans-synaptic co-receptor and ligand abundance were shown to be altered. Since signaling of the Wnt protein Wingless (Wg) is regulated by the heparan sulfate proteoglycan (HSPG) co-receptor Dally-like protein (Dlp) and known to drive the NMJ synaptogenesis, the authors hypothesized that UDP-sugar deficiency triggered changes in the NMJ synaptomatrix glycosylation, including levels of HSPG Dlp, which subsequently affected the Wnt signaling, causing an excessive growth and overelaborated architectural complexity. Mutations in the GALK1- or UDP-glucose dehydrogenase-encoding genes were identified, respectively, as critical environmental and genetic modifiers of behavioral and cellular defects. Double *GALT* and *GALK* mutants or overexpression of UDP-Glc dehydrogenase corrected the glycosylation defects, the NMJ architectural alterations and the movement impairments associated with the *Drosophila* classic galactosemia model. Recently, Daenzer and co-workers reported that the *Drosophila* model did not show phenotypic rescue by knocking out the *GALK1* gene (Daenzer et al. 2016). Additionally, double *GALT* and *GALE* mutants as well as *GALT* and *UGP* mutants displayed severe abnormalities in the coordinated locomotor movement, in NMJ formation, in the synaptomatrix glycosylation and in the Wnt trans-synaptic signaling (Jumbo-Lucioni et al. 2016).

These animal models are able to recapitulate many pathophysiological aspects of classic galactosemia and expand the molecular insights from bacterial and yeast models, bringing new important insights of the underlying pathogenesis.

## Therapeutic approaches in classic galactosemia

In 1935, Mason and Turner described how removing galactose from the diet eliminated the neonatal toxicity and thereafter, dietary galactose restriction became the first recommended therapy for galactosemia that still prevails nowadays.

Considering that classic galactosemia is characterized by deficiency in UDP-hexoses, the potential therapeutic role of uridine was evaluated in a 5-year longitudinal study of galactosemic patients (Manis et al. 1997). Despite no improvements in cognitive function, it remains to be clarified whether uridine was able to enter the cells (Tang et al. 2012).

Aldose reductase inhibition has also been suggested as a therapeutic option, since galactitol is an important pathogenic agent. However, the fact that patients with GALK1 deficiency experience galactitol accumulation but not the broad range of severe long-term complications of classic galactosemia strongly suggests a limited efficacy of aldose reductase inhibitors (Berry 1995).

Another ongoing therapeutic strategy under study concerns the conversion of classic galactosemia into GALK1 deficiency, a milder form of galactosemia in which patients do not accumulate Gal-1-P. GALK1-deficient patients may present cataracts (attributed to galactitol accumulation) and neurological sequelae. In a review from the literature, Bosch et al. reported a frequency of mental retardation in 7% of patients (three out of 43) (Bosch et al. 2003). In a more recent study, approximately 30% of patients (five out of 16) were described as presenting mental retardation, which the authors could not unambiguously attribute to GALK1 deficiency and hypothesized that secondary factors could also take a toll (consanguinity was excluded as a causative agent) (Hennermann et al. 2011). Using recombinant human GALK1 protein, a quantitative high-throughput screening identified a number of small-molecule GALK1 inhibitors, of which the spiro-benzoxazole containing series emerged as lead compounds. These compounds have been further optimized and validated in primary patient fibroblasts, exhibiting reasonable pharmacokinetic properties (Liu et al. 2015; Odejinmi et al. 2011; Tang et al. 2010; Wierenga et al. 2008).

Increased oxidative stress has been reported in the fruit fly model of classic galactosemia, and superoxide dismutase-mimicking manganese-based compounds proved beneficial to GALT-null larvae and adult flies (Jumbo-Lucioni et al. 2014b). Moreover, it has been hypothesized that acetylated anthocyanins derived from purple sweet potato color, previously shown to have a protective effect against galactose toxicity in mice on a high galactose diet (Lu et al. 2010; Zhang et al. 2010), may alleviate galactosemia’s oxidative damage (Timson 2014).

Until recently, the effect of a mutation was assumed to affect only its coding potential. In recent years, however, there has been increasing evidence that both exonic and deep intronic mutations may affect splicing. Mutational analysis of Portuguese patients revealed an intronic variation, c.820 + 13A>G (IVS8 + 13A>G), as the second most frequent mutation. Functional characterization of c.820 + 13A>G revealed it activates the cryptic donor site c.820 + 14_820 + 15, leading to the exonization of the first 13 nucleotides of intron 8, thereby confirming that this intronic variation is actually a disease-causing mutation. Coelho and coworkers employed two locked nucleic acid (LNA) oligonucleotides and the mini-gene approach to successfully restore the splicing profile, thus establishing a proof of concept for the application of antisense therapy for mis-splicing mutations in classic galactosemia (Coelho et al. 2015b).

## Classic galactosemia: where do we stand?

After more than 100 years since the first description of galactosemia, there are still many “clouds gathering over galactosemia” (Anonymous 1982):

- The only currently available treatment, though very effective at preventing or resolving acute and potentially lethal sequelae in the neonatal period, is ineffective in preventing long-term sequelae. Despite early diagnosis and early implementation of therapy, patients still go on to develop burdensome chronic complications.
- Younger siblings of galactosemic patients put on a galactose-restricted diet immediately after birth fail to show a better clinical outcome than the older siblings later in life.
- The reduction in galactose intake by restricting fruit and vegetables seems to be negligible and it has been described that a slightly higher galactose intake may actually improve glycosylation abnormalities. Many countries have now liberalized the therapy, currently advising a lactose-free diet.
- Severe impairment of GALT activity results in the accumulation of galactose metabolites and in UDP-hexoses deficiency. As GALT substrate, Gal-1-P accumulation has been widely pointed out as a key pathogenic agent. Glycosylation abnormalities are extensively reported in classic galactosemia and have also been suggested as an important pathogenic factor.
- Small-molecule GALK1 inhibitors that mitigate Gal-1-P accumulation are being explored as possible therapeutic agents for classic galactosemia and studies on patients’ fibroblasts have shown encouraging results.
- The pathogenic mechanism underlying the severe impairment of GALT activity is protein misfolding and/or aggregation, leading to the recognition of classic galactosemia as a conformational disorder. In recent years, the paradigm that the great majority of inherited diseases are conformational disorders has emerged, which has provided a common framework for the analysis of rather diverse diseases, including Alzheimer’s and Parkinson’s diseases, and many other proteinopathies.
- The most suitable therapeutic strategy for conformational disorders focuses on pharmacological/chemical chaperones, which support the proper folding of protein variants and improve their stability and consequently their activity above a functional threshold. In the specific case of classic galactosemia, a chaperone-based therapy would aim to mitigate GALT misfolding and aggregation, thereby enhancing GALT activity/stability. Considering the molecular heterogeneity of the *GALT* locus, which leads to a high number of protein variants, ideally pharmacological/chemical chaperones should act in a non-mutation-specific way.
- The recently reported crystallographic structure of human GALT provides new insights on the mutations’ effect on GALT structure and function, and will support the design of compounds with pharmacological interest.
- The improvement of patients’ outcome is also hindered by differences in care provided worldwide to galactosemic patients (Jumbo-Lucioni et al. 2012).
- In 2012, an international network of galactosemia—GalNet —was established, aiming at the harmonization and improvement of patients’ outcome and care (Rubio-Gozalbo et al. 2016). It includes professionals from centers in 18 European countries, Israel, Australia, and the USA (www.galactosemianetwork.org). Thus far, a patient Registry has been developed and is currently being implemented across countries; expert- and evidence-based guidelines for treatment and follow-up have been devised, and collaborative research is taking place.

In conclusion, although the outcome for classic galactosemia is still very disappointing at present, there have been encouraging developments in recent years. With the recent growth of knowledge on the molecular basis of galactosemia, there is now a new hope for the development of a truly effective therapy, possibly combining different yet complementary approaches.

## References

[CR1] Anonymous (1982). Clouds over galactosaemia. Lancet.

[CR2] Antshel KM, Epstein IO, Waisbren SE (2004). Cognitive strengths and weaknesses in children and adolescents homozygous for the galactosemia Q188R mutation: a descriptive study. Neuropsychology.

[CR3] Ashino J, Okano Y, Suyama I (1995). Molecular characterization of galactosemia (Type 1) mutations in Japanese. Hum Mutat.

[CR4] Batey LA, Welt CK, Rohr F (2013). Skeletal health in adult patients with classic galactosemia. Osteoporos Int.

[CR5] Belman AL, Moshe SL, Zimmerman RD (1986). Computed tomographic demonstration of cerebral edema in a child with galactosemia. Pediatrics.

[CR6] Berry GT (1995). The role of polyols in the pathophysiology of hypergalactosemia. Eur J Pediatr.

[CR7] Berry GT (2008). Galactosemia and amenorrhea in the adolescent. Ann N Y Acad Sci.

[CR8] Berry GT (2012). Galactosemia: when is it a newborn screening emergency?. Mol Genet Metab.

[CR9] Berry GT, Walter JH (2012) Disorders of galactose metabolism. In: Saudubray JM, van den Berghe G, Walter JH (eds) Inborn metabolic diseases: diagnosis and treatment. Springer, Heidelberg

[CR10] Berry GT, Palmieri M, Gross KC (1993). The effect of dietary fruits and vegetables on urinary galactitol excretion in galactose-1-phosphate uridyltransferase deficiency. J Inherit Metab Dis.

[CR11] Berry GT, Nissim I, Gibson JB (1997). Quantitative assessment of whole body galactose metabolism in galactosemic patients. Eur J Pediatr.

[CR12] Berry GT, Hunter JV, Wang Z (2001). *In vivo* evidence of brain galactitol accumulation in an infant with galactosemia and encephalopathy. J Pediatr.

[CR13] Berry GT, Moate PJ, Reynolds RA (2004). The rate of *de novo* galactose synthesis in patients with galactose-1-phosphate uridyltransferase deficiency. Mol Genet Metab.

[CR14] Beutler E (1991). Galactosemia: screening and diagnosis. Clin Biochem.

[CR15] Bhat PJ (2003). Galactose-1-phosphate is a regulator of inositol monophosphatase: a fact or a fiction?. Med Hypotheses.

[CR16] Bhat M, Haase C, Lee PJ (2005). Social outcome in treated individuals with inherited metabolic disorders: UK study. J Inherit Metab Dis.

[CR17] Bosch AM (2006). Classical galactosaemia revisited. J Inherit Metab Dis.

[CR18] Bosch AM, Bakker HD, Van Gennip AH, Van Kempen JV, Wanders RJA, Wijburg FA (2003). Clinical features of galactokinase deficiency: a review of the literature. J Inherit Metab Dis.

[CR19] Bosch AM, Bakker HD, Wenniger-Prick LJ, Wanders RJ, Wijburg FA (2004). High tolerance for oral galactose in classical galactosaemia: dietary implications. Arch Dis Child.

[CR20] Bosch AM, Grootenhuis MA, Bakker HD, Heijmans HSA, Wijburg FA, Last BF (2004). Living with classical galactosemia: health-related quality of life consequences. Pediatrics.

[CR21] Bosch AM, Maurice-Stam H, Wijburg FA, Grootenhuis MA (2009). Remarkable differences: the course of life of young adults with galactosaemia and PKU. J Inherit Metab Dis.

[CR22] Briones P, Giros M, Martinez V (2001). Second spontaneous pregnancy in a galactosaemic woman homozygous for the Q188R mutation. JIMD.

[CR23] Calderon FR, Phansalkar AR, Crockett DK, Miller M, Mao R (2007). Mutation database for the galactose-1-phosphate uridyltransferase (*GALT*) gene. Hum Mutat.

[CR24] Carney AE, Sanders RD, Garza KR (2009). Origins, distribution and expression of the Duarte-2 (D2) allele of galactose-1-phosphate uridylyltransferase. Hum Mol Genet.

[CR25] Charlwood J, Clayton P, Keir G, Mian N, Winchester B (1998). Defective galactosylation of serum transferrin in galactosemia. Glycobiology.

[CR26] Chen J, Yager C, Reynolds R, Palmieri M, Segal S (2002). Erythrocyte galactose 1-phosphate quantified by isotope-dilution gas chromatography–mass spectrometry. Clin Chem.

[CR27] Chen J, Yager CT, Reynolds RA, Segal S (2002). Identification of galactitol and galactonate in red blood cells by gas chromatography/mass spectrometry. Clin Chim Acta.

[CR28] Clarke JTR (2005). Newborn screening. A clinical guide to inherited metabolic diseases.

[CR29] Coelho AI, Ramos R, Gaspar A (2014). A frequent splicing mutation and novel missense mutations color the updated mutational spectrum of classic galactosemia in Portugal. J Inherit Metab Dis.

[CR30] Coelho AI, Trabuco M, Ramos R (2014). Functional and structural impact of the most prevalent missense mutations in classic galactosemia. Mol Genet Genomic Med.

[CR31] Coelho AI, Trabuco M, Silva M (2015). Arginine functionally improves clinically relevant human galactose-1-phosphate uridylyltransferase (GALT) variants expressed in a prokaryotic model. J Inherit Metab Dis Rep.

[CR32] Coelho AI, Berry GT, Rubio-Gozalbo ME (2015). Galactose metabolism and health. Curr Opin Clin Nutr Metab Care.

[CR33] Coelho AI, Lourenco S, Trabuco M (2015). Functional correction by antisense therapy of a splicing mutation in the GALT gene. Eur J Hum Genet.

[CR34] Coman DJ, Murray D, Byrne JC (2010). Galactosemia, a single gene disorder with epigenetic consequences. Pediatr Res.

[CR35] Coss KP, Byrne JC, Coman DJ (2012). IgG N-glycans as potential biomarkers for determining galactose tolerance in Classical Galactosaemia. Mol Genet Metab.

[CR36] Coss KP, Doran PP, Owoeye C (2013). Classical Galactosaemia in Ireland: incidence, complications and outcomes of treatment. J Inherit Metab Dis.

[CR37] Coss KP, Hawkes CP, Adamczyk B (2014). N-glycan abnormalities in children with galactosemia. J Proteome Res.

[CR38] Crews C, Wilkinson KD, Wells L, Perkins C, Fridovich-Keil JL (2000). Functional consequence of substitutions at residue 171 in human galactose-1-phosphate uridylyltransferase. J Biol Chem.

[CR39] Crome L (1962). A case of galactosaemia with the pathological and neuropathological findings. Arch Dis Child.

[CR40] Cuthbert C, Klapper H, Elsas L (2008). Diagnosis of inherited disorders of galactose metabolism. Curr Protoc Hum Genet.

[CR41] Daenzer JM, Jumbo-Lucioni PP, Hopson ML, Garza KR, Ryan EL, Fridovich-Keil JL (2016). Acute and long-term outcomes in a *Drosophila melanogaster* model of classic galactosemia occur independently of galactose-1-phosphate accumulation. Dis Model Mech.

[CR42] De Jongh S, Vreken PLIJ, Ijlst L, Wanders RJA, Jakobs CAJM, Bakker HD (1999). Spontaneous pregnancy in a patient with classical galactosaemia. JIMD.

[CR43] Decombaz J, Jentjens R, Ith M (2011). Fructose and galactose enhance postexercise human liver glycogen synthesis. Med Sci Sports Exerc.

[CR44] De-Souza EA, Pimentel FS, Machado CM (2014). The unfolded protein response has a protective role in yeast models of classic galactosemia. Dis Model Mech.

[CR45] Douglas HC, Hawthorne DC (1964). Enzymatic expression and genetic linkage of genes controlling galactose utilization in *Saccharomyces*. Genetics.

[CR46] Douglas H, Hawthorne D (1966). Regulation of genes controlling synthesis of the galactose pathway enzymes in yeast. Genetics.

[CR47] Doyle CM, Channon S, Orlowska D, Lee PJ (2010). The neuropsychological profile of galactosaemia. J Inherit Metab Dis.

[CR48] EGS (2003) Galactosaemia in Europe - a comparison. In EGS News (European Galactosaemia Society), p 40

[CR49] Elsas LJ II (2010) Galactosemia. In: Pagon R, Adam MP, Bird TD, Dolan CR, Fong C-T, Smith RJH, Stephens K (eds) Gene reviews. University of Washington, Seattle

[CR50] Elsas LJ, Dembure PP, Langley S, Paulk EM, Hjelm LN, Fridovich-Keil JL (1994). A common mutation associated with the Duarte Galactosemia allele. Am J Hum Genet.

[CR51] Elsas LJ, Langley S, Paulk EM, Hjelm LN, Dembure PP (1995). A molecular approach to galactosemia. Eur J Pediatr.

[CR52] Elsas LJ, Lai K, Saunders CJ, Langley SD (2001). Functional analysis of the human galactose-1-phosphate uridyltransferase promoter in Duarte and LA variant galactosemia. Mol Genet Metab.

[CR53] Facchiano A, Marabotti A (2010). Analysis of galactosemia-linked mutations of GALT enzyme using a computational biology approach. Protein Eng Des Sel.

[CR54] Field TL, Reznikoff WS, Frey PA (1989). Galactose-1-phosphate uridylyltransferase: identification of histidine-164 and histidine-166 as critical residues by site-directed mutagenesis. Biochemistry.

[CR55] Flanagan JM, Tighe O, O’ Neill C, Naughten E, Mayne PD, Croke DT (2004). Identification of sequence variation in the galactose-1-phosphate uridyl transferase gene by dHPLC. Mol Genet Metab.

[CR56] Flanagan JM, McMahon G, Brendan Chia SH (2010). The role of human demographic history in determining the distribution and frequency of transferase-deficient galactosaemia mutations. Heredity (Edinb).

[CR57] Forges T, Monnier-Barbarino P, Leheup B, Jouvet P (2006). Pathophysiology of impaired ovarian function in galactosaemia. Hum Reprod Update.

[CR58] Fridovich-Keil JL, Jinks-Robertson S (1993). A yeast expression system for human galactose-1-phosphate uridylyltransferase. Proc Natl Acad Sci U S A.

[CR59] Fridovich-Keil JL, Walter JH, Valle D, Beaudet AL, Vogelstein B, Kinzler KW, Antonarakis SE, Ballabio A (2008). Galactosemia. The online metabolic and molecular bases of inherited disease.

[CR60] Fridovich-Keil JL, Quimby BB, Wells L, Mazur LA, Elsevier JP (1995). Characterization of the N314D allele of human galactose-1-phosphate uridylyltransferase using a yeast expression system. Biochem Mol Med.

[CR61] Fridovich-Keil JL, Gubbels CS, Spencer JB, Sanders RD, Land JA, Rubio-Gozalbo E (2011). Ovarian function in girls and women with GALT-deficiency galactosemia. J Inherit Metab Dis.

[CR62] Geeganage S, Frey PA (1998). Transient kinetics of formation and reaction of the uridylyl-enzyme form of galactose-1-P uridylyltransferase and its Q168R-variant: insight into the molecular basis of galactosemia. Biochemistry.

[CR63] Geeganage S, Frey PA (1999). Significance of metal ions in galactose-1-phosphate uridylyltransferase: an essential structural zinc and a nonessential structural iron. Biochemistry.

[CR64] Gitzelmann R (1969). Formation of galactose-1-phosphate from uridine diphosphate galactose in erythrocytes from patients with galactosemia. Pediatr Res.

[CR65] Gitzelmann R (1995). Galactose-1-phosphate in the pathophysiology of galactosemia. Eur J Pediatr.

[CR66] Greber-Platzer S, Guldberg P, Scheibenreiter S (1997). Molecular heterogeneity of classical and Duarte Galactosemia: mutation analysis by denaturing gradient gel electrophoresis. Hum Mutat.

[CR67] Gropper SS, Weese SJO, West PA, Gross KC (2000). Free galactose content of fresh fruits and strained fruit and vegetable baby foods. J Am Diet Assoc.

[CR68] Gross KC, Acosta PB (1991). Fruits and vegetables are a source of galactose: implications in planning the diets of patients with galactosaemia. J Inherit Metab Dis.

[CR69] Gross K, Weese S, Johnson J, Gropper S (1995). Soluble galactose content of selected baby food cereals and juices. J Food Compos Anal.

[CR70] Gubbels CS, Land JA, Estela Rubio-Gozalbo M (2008). Fertility and impact of pregnancies on the mother and child in classic galactosemia. Obstet Gynecol Surv.

[CR71] Gubbels CS, Kuppens SM, Bakker JA (2009). Pregnancy in classic galactosemia despite undetectable anti-Mullerian hormone. Fertil Steril.

[CR72] Gubbels CS, Maurice-Stam H, Berry GT (2011). Psychosocial developmental milestones in men with classic galactosemia. J Inherit Metab Dis.

[CR73] Gubbels CS, Welt CK, Dumoulin JC (2013). The male reproductive system in classic galactosemia: cryptorchidism and low semen volume. J Inherit Metab Dis.

[CR74] Haberland C, Perou M, Brunngraber EG, Hof H (1971). The neuropathology of galactosemia: a histopathological and biochemical study. J Neuropathol Exp Neurol.

[CR75] Hansen TWR, Lie SO (1988). Galactosemia - to screen or not to screen?. Pediatrics.

[CR76] Hennermann J, Schadewaldt P, Vetter B, Shin Y, Mönch E, Klein J (2011). Features and outcome of galactokinase deficiency in children diagnosed by newborn screening. J Inherit Metab Dis.

[CR77] Hirokawa H, Okano Y, Asada M, Fujimoto A, Suyama I, Isshiki G (1999). Molecular basis for phenotypic heterogeneity in galactosaemia: prediction of clinical phenotype from genotype in Japanese patients. Eur J Hum Genet.

[CR78] Hoffmann B, Dragano N, Schweitzer-Krantz S (2012). Living situation, occupation and health-related quality of life in adult patients with classic galactosemia. J Inherit Metab Dis.

[CR79] Hughes J, Ryan S, Lambert D (2009). Outcomes of siblings with classical galactosemia. J Pediatr.

[CR80] Jaeken J, Kint J, Spaapen L (1992). Serum lysosomal enzyme abnormalities in galactosaemia. Lancet.

[CR81] Jakobs C, Schweitzer S, Dorland B (1995). Galactitol in galactosemia. Eur J Pediatr.

[CR82] Jensen UG, Brandt NJ, Christensen E, Skovby F, Norgaard-Pedersen B, Simonsen H (2001). Neonatal screening for galactosemia by quantitative analysis of hexose monophosphates using tandem mass spectrometry: a retrospective study. Clin Chem.

[CR83] Jumbo-Lucioni PP, Garber K, Kiel J (2012). Diversity of approaches to classic galactosemia around the world: a comparison of diagnosis, intervention, and outcomes. J Inherit Metab Dis.

[CR84] Jumbo-Lucioni PP, Parkinson W, Broadie K (2014a) Overelaborated synaptic architecture and reduced synaptomatrix glycosylation in a Drosophila classic galactosemia disease model. Dis Model Mech 10.1242/dmm.017137 PMC4257005 25326312

[CR85] Jumbo-Lucioni PP, Ryan EL, Hopson ML (2014). Manganese-based superoxide dismutase mimics modify both acute and long-term outcome severity in a Drosophila melanogaster model of classic galactosemia. Antioxid Redox Signal.

[CR86] Jumbo-Lucioni PP, Parkinson WM, Kopke DL, Broadie K (2016) Coordinated movement, neuromuscular synaptogenesis and trans-synaptic signaling defects in *Drosophila* galactosemia models. Hum Mol Genet 10.1093/hmg/ddw217 PMC5216615 27466186

[CR87] Karadag N, Zenciroglu A, Eminoglu FT (2013). Literature review and outcome of classic galactosemia diagnosed in the neonatal period. Clin Lab.

[CR88] Kaufman F, Kogut M, Donnell G, Goebelsmann U, March C, Koch R (1981). Hypergonadotropic hypogonadism in female patients with galactosemia. New Engl J Med.

[CR89] Kaufman FR, Loro ML, Azen C, Wenz E, Gilsanz V (1993). Effect of hypogonadism and deficient calcium intake on bone density in patients with galactosemia. J Pediatr.

[CR90] Kaufman FR, McBride-Chang C, Manis FR, Wolff J, Nelson L (1995). Cognitive functioning, neurologic status and brain imaging in classical galactosemia. Eur J Pediatr.

[CR91] Kim H, Hartnett C, Scaman C (2007). Free galactose content in selected fresh fruits and vegetables and soy beverages. J Agric Food Chem.

[CR92] Kimonis V (2001). Increased fertility in a woman with classic galactosaemia. JIMD.

[CR93] Koch TK, Schmidt KA, Wagstaff JE, Ng WG, Packman S (1992). Neurologic complications in galactosemia. Pediatr Neurol.

[CR94] Kozak L, Francova H (1999). Presence of a deletion in the 5′ upstream region of the GALT gene in Duarte (D2) alleles. J Med Genet.

[CR95] Kozák L, Francová H, Fajkusová L (1999). Mutation analysis of the GALT gene in Czech and Slovak galactosemia populations: identification of six novel mutations, including a stop codon mutation (X380R). Hum Mutat.

[CR96] Krabbi K, Uudelepp ML, Joost K, Zordania R, Ounap K (2011). Long-term complications in Estonian galactosemia patients with a less strict lactose-free diet and metabolic control. Mol Genet Metab.

[CR97] Kushner RF, Ryan EL, Sefton JM (2010). A Drosophila melanogaster model of classic galactosemia. Dis Model Mech.

[CR98] Lai K, Elsas LJ (2001). Structure-function analyses of a common mutation in blacks with transferase-deficiency galactosemia. Mol Genet Metab.

[CR99] Lai K, Klapa MI (2004). Alternative pathways of galactose assimilation: could inverse metabolic engineering provide an alternative to galactosemic patients?. Metab Eng.

[CR100] Lai K, Langley SD, Singh RH, Dembure PP, Hjelm LN, Elsas LJ (1996). A prevalent mutation for galactosemia among black Americans. J Pediatr.

[CR101] Lai K, Willis AC, Elsas LJ (1999). The biochemical role of glutamine 188 in human galactose-1-phosphate uridyltransferase. J Biol Chem.

[CR102] Lai K, Langley SD, Khwaja FW, Schmitt EW, Elsas LJ (2003). GALT deficiency causes UDP-hexose deficit in human galactosemic cells. Glycobiology.

[CR103] Lai KW, Cheng LY, Cheung AL, WS O (2003). Inhibitor of apoptosis proteins and ovarian dysfunction in galactosemic rats. Cell Tissue Res.

[CR104] Lai K, Elsas LJ, Wierenga KJ (2009). Galactose toxicity in animals. IUBMB Life.

[CR105] Langley SD, Lai K, Dembure PP, Hjelm LN, Elsas LJ (1997). Molecular basis for Duarte and Los Angeles variant galactosemia. Am J Hum Genet.

[CR106] Lebea PJ, Pretorius PJ (2005). The molecular relationship between deficient UDP-galactose uridyl transferase (GALT) and ceramide galactosyltransferase (CGT) enzyme function: a possible cause for poor long-term prognosis in classic galactosemia. Med Hypotheses.

[CR107] Leslie ND (2003). Insights into the pathogenesis of galactosemia. Annu Rev Nutr.

[CR108] Leslie ND, Immerrman EB, Flach JE, Florez M, Fridovich-Keil JL, Elsas LJ (1992). The human galactose-1-phosphate uridyltransferase gene. Genomics.

[CR109] Leslie ND, Yager KL, McNamara PD, Segal S (1996). A mouse model of galactose-1-phosphate uridyl transferase deficiency. Biochem Mol Med.

[CR110] Levy H (1996). Reproductive effects of maternal metabolic disorders: implications for pediatrics and obstetrics. Turk J Pediatr.

[CR111] Levy H, Driscoll S, Porensky R, Wender D (1984) Ovarian failure in galactosaemia. N Engl J Med 310:50 10.1056/nejm198401053100114 6689742

[CR112] Lewis M (2011). The measurement and significance of ionic calcium in milk–a review. Int J Dairy Technol.

[CR113] Lindhout M, Rubio-Gozalbo ME, Bakker JA, Bierau J (2010). Direct non-radioactive assay of galactose-1-phosphate:uridyltransferase activity using high performance liquid chromatography. Clin Chim Acta.

[CR114] Liu G, Hale GE, Hughes CL (2000). Galactose metabolism and ovarian toxicity. Reprod Toxicol.

[CR115] Liu Y, Xia B, Gleason TJ (2012). N- and O-linked glycosylation of total plasma glycoproteins in galactosemia. Mol Genet Metab.

[CR116] Liu L, Tang M, Walsh MJ (2015). Structure activity relationships of human galactokinase inhibitors. Bioorg Med Chem Lett.

[CR117] Lo W, Packman S, Nash S (1984). Curious neurologic sequelae in galactosemia. Pediatrics.

[CR118] Lu J, Wu DM, Zheng YL, Hu B, Zhang ZF (2010). Purple sweet potato color alleviates d‐galactose‐induced brain aging in old mice by promoting survival of neurons via PI3K pathway and inhibiting cytochrome c‐mediated apoptosis. Brain Pathol.

[CR119] Lukac-Bajalo J, Kuzelicki NK, Zitnik IP, Mencej S, Battelino T (2007). Higher frequency of the galactose-1-phosphate uridyl transferase gene K285N mutation in the Slovenian population. Clin Biochem.

[CR120] Lund AM, Hougaard DM, Simonsen H et al (2012) Biochemical screening of 504,049 newborns in Denmark, the Faroe Islands and Greenland—experience and development of a routine program for expanded newborn screening. Mol Genet Metab 107:281–293 10.1016/j.ymgme.2012.06.006 22795865

[CR121] Manga N, Jenkins T, Jackson H, Whittaker DA, Lane AB (1999). The molecular basis of transferase galactosaemia in South African negroids. J Inherit Metab Dis.

[CR122] Manis FR, Cohn LB, McBride-Chang C, Wolff J, Kaufman FR (1997). A longitudinal study of cognitive functioning in patients with classical galactosaemia, including a cohort treated with oral uridine. J Inherit Metab Dis.

[CR123] Marabotti A, Facchiano A (2005). Homology modeling studies on human galactose-1-phosphate uridylyltransferase and on its galactosemia-related mutant Q188R provide an explanation of molecular effects of the mutation on homo-and heterodimers. J Med Chem.

[CR124] Maratha A, Stockmann H, Coss KP et al (2016) Classical galactosaemia: novel insights in IgG N-glycosylation and N-glycan biosynthesis. Eur J Hum Genet 24:976-84 10.1038/ejhg.2015.254 PMC5070905 26733289

[CR125] McCorvie TJ, Gleason TJ, Fridovich-Keil JL, Timson DJ (2013). Misfolding of galactose 1-phosphate uridylyltransferase can result in type I galactosemia. Biochim Biophys Acta.

[CR126] McCorvie T, Kopec J, Pey A (2016). Molecular basis of classic galactosemia from the structure of human galactose 1-phosphate uridylyltransferase. Hum Mol Genet.

[CR127] Mumma JO, Chhay JS, Ross KL, Eaton JS, Newell-Litwa KA, Fridovich-Keil JL (2008). Distinct roles of galactose-1P in galactose-mediated growth arrest of yeast deficient in galactose-1P uridylyltransferase (GALT) and UDP-galactose 4′-epimerase (GALE). Mol Genet Metab.

[CR128] Nelson MD, Wolff JA, Cross CA, Donnel GN, Kaufman FR (1992). Galactosemia: evaluation with MR imaging. Radiology.

[CR129] Ng WG, Bergren WR, Donnel GN (1973). A new variant of galactose-1-phosphate uridyltransferase in man: the Los Angeles variant. Ann Hum Genet.

[CR130] Ng DT, Xu YK, Kaufman FR, Donnel GN (1989). Deficit of uridine diphosphate galactose in galactosaemia. J Inherit Metab Dis.

[CR131] Ning C, Segal S (2000). Plasma galactose and galactitol concentration in patients with galactose-1-phosphate uridyltransferase deficiency galactosemia: determination by gas chromatography/mass spectrometry. Metabolism.

[CR132] Ning C, Fenn PT, Blair IA, Berry GT, Segal S (2000). Apparent galactose appearance rate in human galactosemia based on plasma [^13^C]galactose isotopic enrichment. Mol Genet Metab.

[CR133] Odejinmi S, Rascon R, Tang M, Vankayalapati H, Lai K (2011). Structure-activity analysis and cell-based optimization of human galactokinase inhibitors. ACS Med Chem Lett.

[CR134] Ohlsson A, Nasiell J, von Dobeln U (2007). Pregnancy and lactation in a woman with classical galactosaemia heterozygous for p.Q188R and p.R333W. J Inherit Metab Dis.

[CR135] Ohlsson A, Guthenberg C, von Döbeln U (2012). Galactosemia screening with low false-positive recall rate: the Swedish experience. J Inherit Metab Dis Rep Case Res Rep.

[CR136] Palmieri M, Mazur A, Berry G (1999). Urine and plasma galactitol in patients with galactose-1-phosphate uridyltransferase deficiency galactosemia. Metabolism.

[CR137] Panis B, Forget PP, van Kroonenburgh MJ (2004). Bone metabolism in galactosemia. Bone.

[CR138] Panis B, Forget PP, Nieman FH, van Kroonenburgh MJ, Rubio-Gozalbo ME (2005). Body composition in children with galactosaemia. J Inherit Metab Dis.

[CR139] Panis B, Vermeer C, van Kroonenburgh MJ (2006). Effect of calcium, vitamins K1 and D3 on bone in galactosemia. Bone.

[CR140] Panis B, Gerver WJ, Rubio-Gozalbo ME (2007). Growth in treated classical galactosemia patients. Eur J Pediatr.

[CR141] Petry KG (1991) Characterization of a novel biochemical abnormality in galactosemia: deficiency of glycolipids containing galactose or N-acetylgalactosamine and accumulation of precursors in brain and lymphocytes. Biochem Med Metab Biol 46 10.1016/0885-4505(91)90054-o 1931160

[CR142] Pintor J (2012) Sugars, the crystalline lens and the development of cataracts. Biochem Pharmacol 1:e119

[CR143] Podskarbi T, Kohlmetz T, Gathof BS (1996). Molecular characterization of Duarte-1 and Duarte-2 variants of galactose-l-phosphate uridyltransferase. J Inherit Metab Dis.

[CR144] Pollitt RJ, Green A, McCabe CJ (1997). Neonatal screening for inborn errors of metabolism: cost, yield and outcome. Health Technol Assess Rep.

[CR145] Portnoi PA, MacDonald A (2009). Determination of the lactose and galactose content of cheese for use in the galactosaemia diet. J Hum Nutr Diet 22:400-408 10.1111/j.1365-277X.2009.00948.x 19486453

[CR146] Portnoi PA, Macdonald A (2011). The lactose content of Mini Babybel and suitability for galactosaemia. J Hum Nutr Diet 24:620-621 10.1111/j.1365-277X.2011.01214.x 22023502

[CR147] Portnoi PA, Macdonald A (2016). The lactose and galactose content of cheese suitable for galactosaemia: new analysis. JIMD Reports, 29:85-87 10.1007/8904_2015_520 PMC5059206 26683467

[CR148] Potter NL (2011). Voice disorders in children with classic galactosemia. J Inher Metab Dis.

[CR149] Potter NL, Lazarus JA, Johnson JM, Steiner RD, Shriberg LD (2008). Correlates of language impairment in children with galactosaemia. J Inherit Metab Dis.

[CR150] Potter NL, Nievergelt Y, Shriberg LD (2013). Motor and speech disorders in classic galactosemia. JIMD Rep.

[CR151] Quan-Ma R, Wells HJ, Wells WW, Sherman FE, Egan TJ (1966). Galactitol in the tissues of a galactosemic child. Am J Dis Child.

[CR152] Reichardt JK (1991) Molecular basis of galactosemia: Mutations and polymorphisms in the gene encoding human galactose-1-phosphate uridylyltransferase. Proc Natl Acad Sci U S A 10.1073/pnas.88.7.2633 PMC51292 2011574

[CR153] Riehman K, Crews C, Fridovich-Keil JL (2001). Relationship between genotype, activity, and galactose sensitivity in yeast expressing patient alleles of human galactose-1-phosphate uridylyltransferase. J Biol Chem.

[CR154] Robertson A, Singh RH, Guerrero NV, Hundley M, Elsas LJ (2000). Outcomes analysis of verbal dyspraxia in classic galactosemia. Genet Med.

[CR155] Roe TF, Hallatt JG, Donnell GN, Ng WG (1971). Childbearing by a galactosemic woman. Eur J Pediatr.

[CR156] Ross KL, Davis CN, Fridovich-Keil JL (2004). Differential roles of the Leloir pathway enzymes and metabolites in defining galactose sensitivity in yeast. Mol Genet Metab.

[CR157] Rubio-Gozalbo ME (2002). Bone mineral density in patients with classic galactosaemia. Arch Dis Child.

[CR158] Rubio-Gozalbo ME, Panis B, Zimmermann LJ, Spaapen LJ, Menheere PP (2006). The endocrine system in treated patients with classical galactosemia. Mol Genet Metab.

[CR159] Rubio-Gozalbo ME, Gubbels CS, Bakker JA, Menheere PP, Wodzig WK, Land JA (2010). Gonadal function in male and female patients with classic galactosemia. Hum Reprod Update.

[CR160] Rubio-Gozalbo ME, Bosch AM, Burlina A, Berry GT, Treacy EP, Steering Committee on behalf of all Galactosemia Network representatives (2016) The galactosemia network (GalNet). J Inherit Metab Dis 40:169-170 10.1007/s10545-016-9989-y 27837294

[CR161] Samuels S, Sun SC, Verasestakul S (1976). Normal infant birth in white galactosemic woman. J Med Soc.

[CR162] Sanders RD, Spencer JB, Epstein MP (2009). Biomarkers of ovarian function in girls and women with classic galactosemia. Fertil Steril.

[CR163] Schadewaldt P (2004). Age dependence of endogenous galactose formation in Q188R homozygous galactosemic patients. Mol Genet Metab.

[CR164] Schadewaldt P, Kamalanathan L, Hammen HW, Wendel U (2003). Stable-isotope dilution analysis of galactose metabolites in human erythrocytes. Rapid Commun Mass Spectrom.

[CR165] Schadewaldt P, Kamalanathan L, Hammen H-W, Kotzka J, Wendel U (2014). Endogenous galactose formation in galactose-1-phosphate uridyltransferase deficiency. Arch Physiol Biochem.

[CR166] Schweitzer S, Shin Y, Jakobs C, Brodehl J (1993). Long-term outcome in 134 patients with galactosaemia. Eur J Pediatr.

[CR167] Shah V, Friedman S, Moore AM, Platt BA, Feigenbaum ASJ (2001). Selective screening for neonatal galactosemia: an alternative approach. Acta Paediatr.

[CR168] Shield JP, Wadsworth EJ, MacDonald A (2000). The relationship of genotype to cognitive outcome in galactosaemia. Arch Dis Child.

[CR169] Singh R, Thapa BR, Kaur G, Prasad R (2012). Frequency distribution of Q188R, N314D, Duarte 1, and Duarte 2 GALT variant alleles in an Indian galactosemia population. Biochem Genet.

[CR170] Slepak T, Tang M, Addo F, Lai K (2005). Intracellular galactose-1-phosphate accumulation leads to environmental stress response in yeast model. Mol Genet Metab.

[CR171] Spencer JB, Badik JR, Ryan EL (2013). Modifiers of ovarian function in girls and women with classic galactosemia. J Clin Endocrinol Metab.

[CR172] Sturiale L, Barone R, Fiumara A (2005). Hypoglycosylation with increased fucosylation and branching of serum transferrin N-glycans in untreated galactosemia. Glycobiology.

[CR173] Suzuki M, West C, Beutler E (2001). Large-scale molecular screening for galactosemia alleles in a pan-ethnic population. Hum Genet.

[CR174] Tang M, Wierenga K, Elsas LJ, Lai K (2010). Molecular and biochemical characterization of human galactokinase and its small molecule inhibitors. Chem Biol Interact.

[CR175] Tang M, Odejinmi SI, Vankayalapati H, Wierenga KJ, Lai K (2012). Innovative therapy for classic galactosemia - tale of two HTS. Mol Genet Metab.

[CR176] Tang M, Siddiqi A, Witt B et al (2014) Subfertility and growth restriction in a new galactose-1 phosphate uridylyltransferase (GALT) - deficient mouse model. Eur J Hum Genet 10.1038/ejhg.2014.12 PMC4169538 24549051

[CR177] Tedesco TA, Morrow G, Mellman WJ (1972). Normal pregnancy and childbirth in a galactosemic woman. Eur J Pediatr.

[CR178] Thoden JB, Ruzicka FJ, Frey PA, Rayment I, Holden HM (1997). Structural analysis of the H166G site-directed mutant of galactose-1-phosphate uridylyltransferase complexed with either UDP-glucose or UDP-galactose: detailed description of the nucleotide sugar binding site. Biochemistry.

[CR179] Timmers I, van den Hurk J, Di Salle F, Rubio-Gozalbo ME, Jansma BM (2011). Language production and working memory in classic galactosemia from a cognitive neuroscience perspective: future research directions. J Inherit Metab Dis.

[CR180] Timmers I, Jansma BM, Rubio-Gozalbo ME (2012). From mind to mouth: event related potentials of sentence production in classic galactosemia. PLoS One.

[CR181] Timmers I, Hurk Jvd, Hofman PA et al (2015) Affected functional networks associated with sentence production in classic galactosemia. Brain Res 1616:166-176 10.1016/j.brainres.2015.05.007 25979518

[CR182] Timmers I, Zhang H, Bastiani M, Jansma BM, Roebroeck A, Rubio-Gozalbo E (2015). Assessing white matter microstructure in classic galactosemia using neurite orientation dispersion and density imaging. J Inherit Metab Dis.

[CR183] Timson DJ (2014) Purple sweet potato colour—a potential therapy for galactosemia? Int J Food Sci Nutr 65:391–393 10.3109/09637486.2013.860586 24279733

[CR184] Timson DJ, Reece RJ (2003). Identification and characterisation of human aldose 1-epimerase. FEBS Lett.

[CR185] Trbušek M, Francova H, Kozak L (2001). Galactosemia: deletion in the 5′ upstream region of the GALT gene reduces promoter efficiency. Hum Genet.

[CR186] Van Calcar S, Bernstein L, Rohr F, Scaman C, Yannicelli S, Berry G (2014). A re-evaluation of life-long severe galactose restriction for the nutrition management of classic galactosemia. Mol Genet Metab.

[CR187] Van Calcar S, Bernstein L, Rohr F, Yannicelli S, Berry G, Scaman C (2014). Galactose content of legumes, caseinates, and some hard cheeses: implications for diet treatment of classic galactosemia. J Agric Food Chem.

[CR188] van Erven B, Gubbels CS, van Golde RJ (2013). Fertility preservation in female classic galactosemia patients. Orphanet J Rare Dis.

[CR189] van Erven B, Welling L, van Calcar SC et al (2017) Bone health in classic galactosemia: systematic review and meta-analysis. JIMD Rep. doi:10.1007/8904_2016_28 10.1007/8904_2016_28 PMC5585100 27995581

[CR190] Wadelius C, Lagerkvist A, Molin A-K, Larsson A, Doebeln UV (1993). Galactosemia caused by a point mutation that activates cryptic donor splice site in the galactose-1-phosphate uridyltransferase gene. Genomics.

[CR191] Waggoner DD, Buist NRM, Donnel GN (1990). Long-term prognosis in galactosaemia: results of a survey of 350 cases. J Inherit Metab Dis.

[CR192] Waisbren SE, Norman TR, Schnell RR, Levy HL (1983). Speech and language deficits in early-treated children with galactosemia. J Pediatr.

[CR193] Waisbren SE, Read CY, Ampola M (2002). Newborn screening compared to clinical identification of biochemical genetic disorders. J Inherit Metab Dis.

[CR194] Waisbren SE, Potter NL, Gordon CM (2012). The adult galactosemic phenotype. J Inherit Metab Dis.

[CR195] Wang ZJ, Berry GT, Dreha SF, Zhao H, Segal S, Zimmerman RA (2001). Proton magnetic resonance spectroscopy of brain metabolites in galactosemia. Ann Neurol.

[CR196] Wedekind JE, Frey PA, Rayment I (1995). Three-dimensional structure of galactose-1-phosphate uridylyltransferase from *Escherichia coli* at 1.8 Å resolution. Biochemistry.

[CR197] Wedekind JE, Frey PA, Rayment I (1996). The structure of nucleotidylated histidine-166 of galactose-1-phosphate uridylyltransferase provides insight into phosphoryl group transfer. Biochemistry.

[CR198] Wehrli SL, Berry GT, Palmieri M, Mazur A, Elsas LJ, Segal S (1997). Urinary galactonate in patients with galactosemia: quantitation by nuclear magnetic resonance spectroscopy. Pediatr Res.

[CR199] Welling L, Bernstein LE, Berry GT et al (2016) International clinical guideline for the management of classical galactosemia: diagnosis, treatment, and follow-up. J Inherit Metab Dis 40:171-176 10.1007/s10545-016-9990-5 PMC5306419 27858262

[CR200] Wells L, Fridovich-Keil JL (1997). Biochemical characterization of the S135L allele of galactose-1-phosphate uridylyltransferase associated with galactosaemia. J Inherit Metab Dis.

[CR201] Wierenga KJ, Lai K, Buchwald P, Tang M (2008). High-throughput screening for human galactokinase inhibitors. J Biomol Screen.

[CR202] Wong L-J, Frey PA (1974). Galactose-1-phosphate uridylyltransferase Isolation of a uridylyl-enzyme intermediate. J Biol Chem.

[CR203] Wong L-J, Frey PA (1974). Galactose-1-phosphate uridylyltransferase. Rate studies confirming a uridylyl-enzyme intermediate on the catalytic pathway. Biochemistry.

[CR204] Wood IS, Trayhurn P (2003). Glucose transporters (GLUT and SGLT): expanded families of sugar transport proteins. Br J Nutr.

[CR205] Yager CT, Chen J, Reynolds R, Segal S (2003). Galactitol and galactonate in red blood cells of galactosemic patients. Mol Genet Metab.

[CR206] Yager C, Wehrli S, Segal S (2006). Urinary galactitol and galactonate quantified by isotope-dilution gas chromatography–mass spectrometry. Clin Chim Acta.

[CR207] Zekanowski C, Radomyska B, Bal J (1999). Molecular characterization of Polish patients with classical galactosaemia. J Inherit Metab Dis.

[CR208] Zhang ZF, Lu J, Zheng YL (2010). Purple sweet potato color protects mouse liver against D-galactose-induced apoptosis via inhibiting caspase-3 activation and enhancing PI3K/Akt pathway. Food Chem Toxicol.

